# The potential roles of stress‐induced phosphoprotein 1 and connexin 43 in rats with reperfusion arrhythmia

**DOI:** 10.1002/iid3.852

**Published:** 2023-10-03

**Authors:** Li An, Hong Gao, Yi Zhong, Yanqiu Liu, Ying Cao, Jing Yi, Xiang Huang, Chunlei Wen, Rui Tong, Zhijun Pan, Xu Yan, Meiyan Liu, Shengzhao Wang, Hao Wu, Tingju Hu

**Affiliations:** ^1^ School of Anesthesiology Guizhou Medical University Guiyang Guizhou China; ^2^ Department of Anaesthesiology Affiliated Hospital of Guizhou Medical University Guiyang Guizhou China; ^3^ Translational Medicine Research Center Guizhou Medical University Guiyang Guizhou China; ^4^ Department of Anesthesiology Guizhou Hospital of The 1st Affiliated Hospital, Sun Yat‐sen University Guiyang Guizhou China; ^5^ Department of Anesthesiology Guiyang Fourth People's Hospital Guiyang Guizhou China; ^6^ Department of Anesthesiology Guiyang Second People's Hospital Guiyang Guizhou China; ^7^ Department of Anesthesiology Children's Hospital of Guiyang Maternal and Child Health Hospital Guiyang Guizhou China

**Keywords:** Connexin 43, HSP70, HSP90, postischemic arrhythmias reperfusion arrhythmia, Rat, stress‐induced phosphoprotein 1, Ubiquitination

## Abstract

**Objective:**

Connexin 43 (Cx43) is a critical gene for maintaining myocardial homeostasis. Interestingly, Cx43 and stress‐induced phosphoprotein 1 (STIP1) were recorded to be lowly expressed in ischemia/reperfusion (I/R). However, their impacts on reperfusion arrhythmia (RA) remain to be explored. Our study aimed to find out the related underlying mechanisms.

**Methods:**

After the establishment of an isolated heart model through Langendorff perfusion, the heart rate, conduction activation time, conduction velocity, and conduction direction of the left ventricle were evaluated, along with the apoptotic rate detection in the collected myocardial tissues. After the construction of a hypoxia/reoxygenation (H/R)‐induced cellular model, cell apoptosis, intercellular communication, cell viability, and the content of reactive oxygen species, superoxide dismutase, malondialdehyde, and lactic dehydrogenase were measured. The expression of Cx43 and STIP1 was determined in both rat heart and cell models. The bindings of STIP3 and Cx43 to  heat shock protein 90 (HSP90) and heat shock protein 70 (HSP70) were verified.

**Results:**

Relative to the corresponding controls, Cx43 and STIP1 were decreased in myocardial tissues of RA rats and H/R‐stimulated H9C2 cells, where Cx43‐binding HSP70 and HSP90 were respectively increased and decreased, and ubiquitination level of Cx43 was enhanced. STIP1 overexpression promoted protein expression of Cx43, intercellular communication, and cell viability, and reduced cell apoptosis and oxidative stress in H/R‐stimulated H9C2 cells.

**Conclusion:**

STIP1 promoted Cx43 expression to improve intercellular communication and reduce oxidative stress in H/R‐stimulated H9C2 cells.

## Graphical abstract

Notes: Cx43 and STIP1 expression was decreased in reperfusion arrhythmia. Cx43 bound to HSP90 to inhibit Cx43 ubiquitination, thereby improving reperfusion arrhythmia. However, the relationship between STIP1 (as a co‐chaperone) and Cx43 needs further experimental verification.

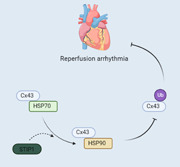

## 1 INTRODUCTION

Myocardial ischemia/reperfusion (I/R) injury (MIRI) refers to a phenomenon wherein the restoration of blood supply to the myocardium after ischemia does not improve cardiac function but leads to secondary tissue damage.^1^ MIRI can prolong action potential duration and QT interval, and these electrophysiological changes increase the risk of the myocardium to arrhythmia.^2^ The most common manifestations of reperfusion arrhythmia (RA) are ventricular tachycardia and ventricular fibrillation (VF), which can lead to hemodynamic disorders and even sudden cardiac death.^3^ Reperfusion therapy‐related arrhythmia has conferred an independent mortality risk factor.^4^ Therefore, how to alleviate susceptibility to RA and protect myocardial functions effectively is always a hot spot in clinical research.

Electrophysiological remodeling during I/R‐related arrhythmia is, in part, owing to cardiac gap junction (GJ) (fluorescence) uncoupling, and the eventual cellular injury can be determined by the acute effects of the passage of cell‐survival or apoptotic signals through open GJ channels between contiguous myocytes.^5^ Connexin 43 (Cx43), a member of the connexins, is a special GJ protein that is most abundant on the cell membrane of mammalian ventricular cardiomyocytes.^6^ Hemichannels are often regarded as “pathologic pores” that open abnormally in response to cellular stress.^7^, ^8^ If the activation of hemichannels was uncontrolled, significant changes may be caused in cardiomyocyte homeostasis, leading to dysfunction and ultimately irreversible injury in the heart.^9^ Increasing evidence showed that preventing the opening of Cx43 hemichannels had a protective effect against I/R injury.^10^, ^11^ Importantly, Andelova et al. found that exposure to constant light contributed to damage of cardiac Cx43 channels‐mediated electrical communication in rats, thereby increasing the risk of malignant cardiac arrhythmias.^12^ In addition, a previous study showed that Cx43 was involved in I/R‐induced arrhythmias and cardiomyocyte apoptosis.^13^ These studies indicate that Cx43 is essential for maintaining cardiomyocyte homeostasis.

A mechanistic study proposed that Cx43 bound to heat shock protein 90 (HSP90) and participated in mitochondrial damage in embryonic stem cell‐derived cardiomyocytes.^14^ In isoproterenol‐induced rat models, T89 promoted Cx43 expression by inhibiting the expression of heat shock protein 70 (HSP70)/HSP40, thus alleviating cardiac hypertrophy.^15^ Rodriguez‐Sinovas et al. found that HSP90 bound to Cx43 and promoted the mitochondrial translocation of Cx43 by TOM20 pathway to exert its cardioprotective functions.^16^ In addition, our pilot study predicted a possible interaction between stress‐induced phosphoprotein 1 (STIP1) and HSP90 through the string database. STIP1 is an adaptor protein that assists in the transfer of client proteins from HSP70 to HSP90 by binding both HSP90 and substrate‐bound HSP70, which was identified to be a widely studied helper molecular chaperone.^17^, ^18^ A previous study demonstrated that STIP1 alleviated I/R‐induced neuronal injury and inflammation in rat spinal cords and mouse microglial cells.^19^ However, studies about the mechanisms and effects of STIP1 in RA are rare. We hypothesized that STIP1 might control RA development via Cx43. Hence, our study probed the specific role and mechanism of the ST1P1/Cx43 in RA with the use of an established isolated Langendorff‐perfused rat heart model and hypoxia/reoxygenation (H/R) cellular model, hoping to help attenuate the risk of I/R‐induced arrhythmias.

## 2 MATERIALS AND METHODS

### 2.1 Cell culture

Rat cardiomyocytes (H9C2; GNR 5) obtained from the National Collection of Authenticated Cell Cultures (China) were maintained in Dulbecco's modified eagle medium (C11995500BT, GIBCO) containing 10% fetal bovine serum at 37°C with 5% CO_2_. When cell growth density reached 80%–90%, cell passage was carried out at a ratio of 2:1.

### 2.2 A cellular model of H/R and transfection

The H/R injury model was conducted in H9C2 cells to simulate MIRI. Briefly, H9C2 cells at the logarithmic phase were divided into negative control (NC), H/R, NC + short hairpin (sh)‐NC, NC + sh‐HSP90, H/R + sh‐NC, H/R + sh‐HSP90, H/R + OE‐NC, and H/R + OE‐STIP1 groups.

HSP90 knockdown vector (sh‐HSP90), STIP1 overexpression vector (OE‐STIP1), and their NCs (sh‐NC, OE‐NC) were purchased from GenePharma. Cell transfection was performed based on the instruction on the Lipofectamine 2000 kit (Invitrogen). Follow‐up experiments were conducted 48 h after the transfection.

Cells in the H/R group were cultured in an anaerobic chamber (95% N_2_, 5% CO_2_) for 1 h, followed by 4 h of reoxygenation (95% O_2_, 5% CO_2_). In the NC group, cells were cultured normally for 5 h.

### 2.3 Quantitative reverse transcription‐polymerase chain reaction (qRT‐PCR)

Total RNA was extracted by Trizol (16096020, Thermo Fisher Scientific). RNA samples with the required concentration and purity were diluted and synthesized into complementary DNA in accordance with the specifications of the reverse transcription kit (TaKaRa). Relative gene expression was evaluated on a LightCycler 480 PCR instrument (Roche) based on the user manual of the fluorescence quantitative PCR kit (SYBR Green Mix, Roche). The reaction conditions were set as follows: pre‐denaturation at 95°C for 5 min; 35 cycles of denaturation at 95°C for 10 s, annealing at 56°C for 10 s, and extension at 72°C for 20 s. Data were acquired from three independent experiments and analyzed with the 2^−ΔΔCt^ method (ΔΔCt = experimental group [Ct target gene − Ct internal control] − control group [Ct target gene − Ct internal control]), having β‐actin as the internal control. The primer sequences of STIP1, Cx43, and β‐actin are synthesized by Sangon Biotechnology Co., Ltd. and shown in Table 1.

**Table 1:** Primer sequences used for quantitative reverse transcription‐polymerase chain reaction analysis.

Name of primer	Sequences
STIP1‐F	5′‐CCTTGCGCTTCCTAGGTGAA‐3′
STIP1‐R	5′‐CAACCGTCCTCATACGCCTT‐3′
Cx43‐F	5′‐TTCATTGGGGGAAAGGCGTG‐3′
Cx43‐R	5′‐CGCCAAAGTTGGTGGAACTC‐3′
β‐Actin‐F	5′‐ACCCGCGAGTACAACCTTCT‐3′
β‐Actin‐R	5′‐GCCGTGTTCAATGGGGTACT‐3′

*Note*: F, forward; R, reverse.

Abbreviation: STIP1, stress‐induced phosphoprotein 1.

### 2.4 Western blotting

Radio‐Immunoprecipitation assay (RIPA) (containing phenylmethylsulfonyl fluoride) lysis was used for extraction of total protein from tissues or cells. After 30 min of incubation on ice and 10 min of centrifugation (8000*g*, 4°C), the supernatant was collected for protein concentration detection with a bicinchoninic acid kit. Next, 50 μg protein was dissolved in 2× sodium dodecyl sulfate (SDS) loading buffer and boiled at 100°C for 5 min, followed by SDS‐polyacrylamide gel electrophoresis. Then the protein was transferred onto a polyvinylidene fluoride membrane, after which the membrane was blocked in 5% skim milk for an hour at room temperature. Afterward, the membrane was incubated at 4°C overnight with primary antibodies from Abcam against STIP1 (ab126753, 1:10,000), HSP90 (ab59459, 1:500), Cx43 (ab11370, 1:2000), and β‐actin (ab8226, 1:2000). After washing, horseradish peroxidase (HRP) labeled goat anti‐rabbit immunoglobulin G (IgG) (1:5000, Beijing ComWin Biotechnology Co., Ltd.) was appended to the membrane for 2 h of incubation, followed by treatment with electrochemical luminescence solution for color development. Finally, the protein bands were scanned and analyzed on a gel imager, and gray quantification of the western blot images was analyzed by the software Image Pro Plus 6.0 (Media Cybernetics). Each experiment was repeated three times.

### 2.5 Cell counting kit 8 (CCK‐8)

Cell viability was evaluated using a CCK‐8 kit (MedChemExpress). Briefly, cells in each group were digested with 0.25% trypsin, and the cell concentration was adjusted to 5 × 10^4^ cells/mL. Next, 100 μL cell suspensions were seeded onto each well of the 96‐well plate for overnight incubation, with three replicate wells for each group. After that, the cells were added with 10 µL CCK‐8 reagent and incubated for another 4 h. An Envision Multilabel Reader (PerkinElmer) was used to determine cell absorbance at 450 nm. Each assay was performed in triplicate.

### 2.6 Flow cytometry

According to the instructions of the Annexin V‐FITC/propidium iodide (PI) cell apoptosis detection kit (TransGen Biotech), cell apoptosis was monitored. Cells were maintained in a 37°C incubator with 5% CO_2_. Afterward, they were collected and washed twice in phosphate buffer saline (PBS) solution, centrifuged, and suspended in 200 μL binding buffer. Later, 10 μL Annexin V‐FITC and 5 μL PI were gently mixed with the cell suspension for 15 min of reaction in the dark. After addition of 300 μL binding buffer, the apoptotic rate was tested by a flow cytometer (BD Biosciences) at the excitation wavelength of 488 nm. Three independent experiments were carried out.

### 2.7 Reactive oxygen species (ROS) in cardiomyocytes

ROS levels in cardiomyocytes were determined by 2′,7′‐dichlorodihydrofluorescein (DCFH‐DA) according to the instructions of the ROS assay kit (HY‐D0940, MedChemExpress). In brief, after cells were cultured in 6‐well plates, the culture medium was discarded, followed by addition of 1 mL DCFH‐DA (10 μmol/L) in each well for 20 min of incubation (37°C) in the dark. After washing thrice with serum‐free culture medium, an inverted fluorescence microscope was used for qualitative observation, and a flow cytometer for detection of fluorescence intensity of each group (excitation wavelength: 488 nm; emission wavelength: 525 nm). The fluorescence intensity was used to evaluate intercellular ROS levels.

### 2.8 Enzyme‐linked immunosorbent assay (ELISA)

The levels of superoxide dismutase (SOD), malondialdehyde (MDA), and lactic dehydrogenase (LDH) were determined by ELISA kits (R&D). Specifically, the samples were added to an ELISA plate and wrapped before overnight incubation at room temperature. After the culture solution was removed, the samples were washed thrice with PBS for 5 min each. Next, 5% bovine serum albumin (BSA) blocking solution was added (100 μL/well) for 1 h of incubation, and then a primary antibody diluted by PBS (containing 5% BSA) was added to the 96‐well plate (100 μL per well) and incubated for 3 h. After washing, the plate was appended with a 5% BSA‐PBS‐diluted HRP‐labeled secondary antibody for an hour of incubation. After that, the plate was washed with PBS, and 10 μL of the substrate was added and cultured with the samples at 37°C for 10–15 min. Finally, the absorbance value of each sample was measured at 450 nm. Each experiment was repeated three times.

### 2.9 Scrape‐loading and dye transfer (SLDT)

Under aseptic conditions, H9C2 cells undergoing 30 min culture with Gap19 (200 μM)^9^ were inoculated in a six‐well plate and placed on an ultra‐clean table with the culture medium absorbed. After washing three times with 0.1 mmol/L PBS (preheated at 37°C), the liquid was absorbed away and cells were gently scratched with a surgical blade. Next, 2 mL of 0.05% lucifer yellow cadaverine (preheated at 37°C) was added to each well. Eight minutes later, the dye was absorbed and the cells were rinsed with PBS, after which the excess solution and dissociative dye were removed. Subsequently, cells were fixed with 1% paraformaldehyde and the transfer of the lucifer yellow dye was observed under the excitation of 488 nm laser with an inverted fluorescence microscope. Semi‐quantitative observation: the distance of lucifer transmission from cells in the scraped area to adjacent cells was observed for semi‐quantitative detection, that is, the number of cell rows of lucifer transferring from primary loaded cells to contiguous cells (0: Lucifer fluorescence was limited to a single row of primary loaded cells; 1: Lucifer fluorescence was transferred from a single row of cells in the scraped area to an adjacent row of cells; 2: Lucifer fluorescence was transferred from one row of cells in the scraped area to two adjacent rows of cells; 3: Lucifer was transferred from a single row of cells in the scraped area to three adjacent rows; 4: Lucifer was transmitted from a single row of cells in the scraped area to cells within four adjacent rows or further away). A total of five wells in the plate were observed and the results were averaged.

### 2.10 Fluorescence co‐localization

Twenty‐four‐well plates were covered with 5% Poly‐d‐Lysine (300–400 μL per well), and H9C2 cells were inoculated on the plates at a density of 3 × 10^4^ cells/mL. H9C2 cells were fixed with 4% paraformaldehyde, blocked with 10% NGS, permeabilized with 0.1% TritonX100, and incubated overnight with rabbit anti‐rat Cx43 (ab235282, Abcam) plus mouse anti‐rat HSP70 (ab2787, Abcam) or anti‐Cx43 plus mouse anti‐rat HSP90 (ab59459, Abcam). Later, the cells were incubated (in the dark, 60 min) with goat anti‐rabbit IgG Alexa Fluor® 488 (ab150077, Abcam) and goat anti‐mouse IgG Alexa Fluor® 647 (ab150115, Abcam), stained by 4′,6‐diamidino‐2‐phenylindole, and then sealed. Finally, the slides were observed under an Olympus FV‐1000 confocal laser microscope.

### 2.11 Co‐immunoprecipitation (Co‐IP)

Cells were lysed with precooled RIPA lysis and 15‐min centrifugation (at 14,000*g* and 4°C) was performed. Then the supernatant was removed to a new tube. Anti‐STIP1 (ab126753, 1:20, Abcam), anti‐Cx43 (ab235585, 1:30, Abcam), anti‐HSP70 (ab2787, 1:50, Abcam), or anti‐HSP90 (ab59459, 1:50, Abcam) was added to 1 mL cell lysate, and IgG antibody (ab172730, 1:100, Abcam) was added to the NC group. The antigen‐antibody mixture was slowly shaken on a shaker overnight at 4°C or for 2 h at room temperature. After that, the mixture was added with 100 μL Protein A/G agarose beads (prepared in PBS with a concentration of 50%) and incubated at 4°C overnight or for 1 h at room temperature. The agarose bead‐antigen‐antibody complex was obtained after centrifugation at 14,000 rpm for 5 s. Next, the complex was rinsed three times with precooled RIPA (800 μL) and suspended with 2× loading buffer (60 μL). The sample was boiled for 5 min and centrifuged to collect the remaining agarose beads, and the supernatant was boiled again for 5 min and then electrophoresed. Protein expression was measured by western blotting.

### 2.12 Detection of Cx43 ubiquitination by Co‐IP

H9C2 cells were transfected with Myc‐Cx43 (Genomeditech) and HA‐ubiquitin (Genomeditech) using LipoFiter™ transfection reagent (HANBIO). Next, the cells were treated with 20 μM proteasome inhibitor MG132 for 6 h, washed twice with precooled PBS, and then lysed with RIPA lysis. Cytoplasmic proteins obtained from the lysate by centrifugation were incubated with anti‐Cx43 overnight. The antigen‐antibody mixture was added with 100 μL Protein A/G agarose beads on a shaker at 4°C for 4 h, followed by washing three times with lysis buffer and boiling in 2× SDS loading buffer. The eluted protein was detected by western blotting with an anti‐ubiquitin antibody (ab134953, 1:2000, Abcam).

### 2.13 Isolated Langendorff‐perfused heart model in rats

Sixteen healthy and clean male Sprague Dawley rats (weighing 250–350 g, 2–3 months old) were selected for this study. All rats were fed in specific pathogen‐free sterile laminar flow chambers at a constant temperature of 22°C–26°C and a constant humidity of 55 ± 5% with free access to food and water. All experiments were approved by the Animal Ethics Committee of the Affiliated Hospital of Guizhou Medical University and conducted in accordance with the ethical requirements of experimental animals.

All rats were divided into control and I/R groups (*n* = 8/group). For the establishment of an isolated Langendorff‐perfused rat heart model, we followed the previous methods.^20^ Specifically, rats were injected intraperitoneally with 3125 U/kg heparin anticoagulation (3%). Ten minutes later, the rats were anesthetized by intraperitoneal injection of 1% pentobarbital sodium (40 mg/kg). After the anesthesia was fully effective, the rats were fixed and the xiphoid was lifted with hemostatic forceps. The anterior chest wall was removed with surgical scissors along the lower margin and lateral wall of the sternum. The tissues around the heart were isolated, the pericardium was opened, and the vessels at the heart bottom were disassociated. The isolated hearts were then quickly placed in 4°C Krebs‐Henseleit (K‐H) solution (mmol/L: NaCl 118, KCl 4.5, CaCl_2_ 1.26, MgSO_4_·7H_2_O 1.22, KH_2_PO_4_ 1.18, NaHCO_3_ 24.99, and C_6_H_12_O_6_ 11.1; pH = 7.4), after which the remaining blood in the aortae was rinsed and the tissues were trimmed to expose the aortae. Subsequently, the aortae were attached to the Langendorff perfusion device (IH‐SR, 844; Germany HUGO SACHS ELEKTRONIK‐HARVARD APPA RATUS GmbH D‐79232 March‐Hugstetten). The depth of aortic intubation was >2 mm from the aortic valve. K‐H solution was preperfused into the hearts under the conditions of 95% O_2_‐5% CO_2_ with constant pressure (8.65 kpa) and temperature (36.5°C–37.5°C) before noncyclic retrograde perfusion. The isolated Langendorff‐perfused heart model was identified successful once the isolated heart returned to normal rhythm (heart rate >180 times/min) within 10 min of balanced perfusion of the K‐H solution. During the experiment, unqualified samples were excluded in time and new rats were added to ensure consistent sample size in each group.

**Figure 1:**
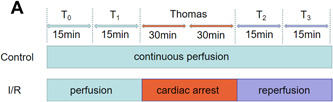
The scheme of the animal model establishment. T_0_, balanced perfusion for 15 min; T_1_, continuous perfusion for 15 min; T_2_, reperfusion for 15 min; T_3_, reperfusion for 30 min. I/R, ischemia reperfusion.

After identification of the model, 16 hearts were selected and randomly divided into two groups by the method of random numbers (*n* = 8 per group). In the control group, rats were continuously perfused with K‐H solution (37°C) for 120 min. In the I/R group, after perfusion of K‐H solution (37°C) for 30 min, Thomas cardioplegic solution (mmol/L: NaCl 110, KCl 16.1, MgCl_2_ 15.96, CaCl_2_ 1.26, CaCl_2_ 9.99; pH = 7.8) (4°C, 20 mL/kg) was injected through the aortic root to cause cardiac arrest for 60 min. After cardiac arrest, the perfusion of K‐H solution was stopped and the hearts were protected with Thomas solution (4°C). After the cardiac arrest for 30 min, 10 mL/kg Thomas solution (4°C) was reperfused. Sixty minutes after the cardiac arrest, K‐H solution (37°C) was reperfused for 30 min. The establishment of the animal model is shown in Figure 1.

### 2.14 Electrical conduction of ventricular muscle anterior wall by microelectrode array

After continuous perfusion for 15 min, the 64 matrix electrode was placed in the anterior wall of the left ventricle, and the reference electrode was placed at the root of the aorta. Then the electrode wire was linked with the MappingLab matrix electrophysiological mapping system. According to the collected local field potential, the matrix electrode was adjusted to fit the heart, and the local electrical conduction of the left ventricle was recorded. The heart rate, conduction velocity, and electric conduction figure were recorded after balanced perfusion for 15 min (T_0_), continuous perfusion for 15 min (T_1_), and reperfusion for 15 min (T_2_) and 30 min (T_3_). The time for restoration of spontaneous heartbeat, as well as the type and duration of arrhythmia and arrhythmia score within 30 min of reperfusion, was recorded and observed.

### 2.15 Collection of myocardial tissues

At the end of reperfusion, the left ventricular myocardial tissues were rapidly collected and placed in liquid nitrogen for cryopreservation. After that, the myocardial tissues were transferred to a refrigerator (−80°C) for subsequent detection.

### 2.16 Terminal deoxynucleotidyl transferase‐mediated dUTP nick‐end labeling (TUNEL) staining

After paraffin embedding and sectioning, apoptosis in cardiac tissues was measured using a TUNEL kit (DeadEnd™ Colorimetric TUNEL System, 000086338, Promega). Image‐pro software was used for image analysis, and six fields were randomly selected from apoptotic positive areas of each image to calculate the apoptotic index (apoptotic index = the number of apoptotic cells/total number of cells × 100%). Each assay was repeated three times.

### 2.17 Statistical analysis

Statistical analysis was conducted using GraphPad prism7 software and all data were shown as mean ± standard deviation. *T*‐test was used to compare data between two groups and one‐way analysis of variance was employed for comparisons among multiple groups with Tukey's multiple comparisons test used for post hoc analysis. A *p* < .05 was thought to have statistical significance. All experiments were run in triplicate.

## 3 RESULTS

### 3.1 I/R induced RA and cardiomyocyte apoptosis in the isolated heart model

**Figure 2:**
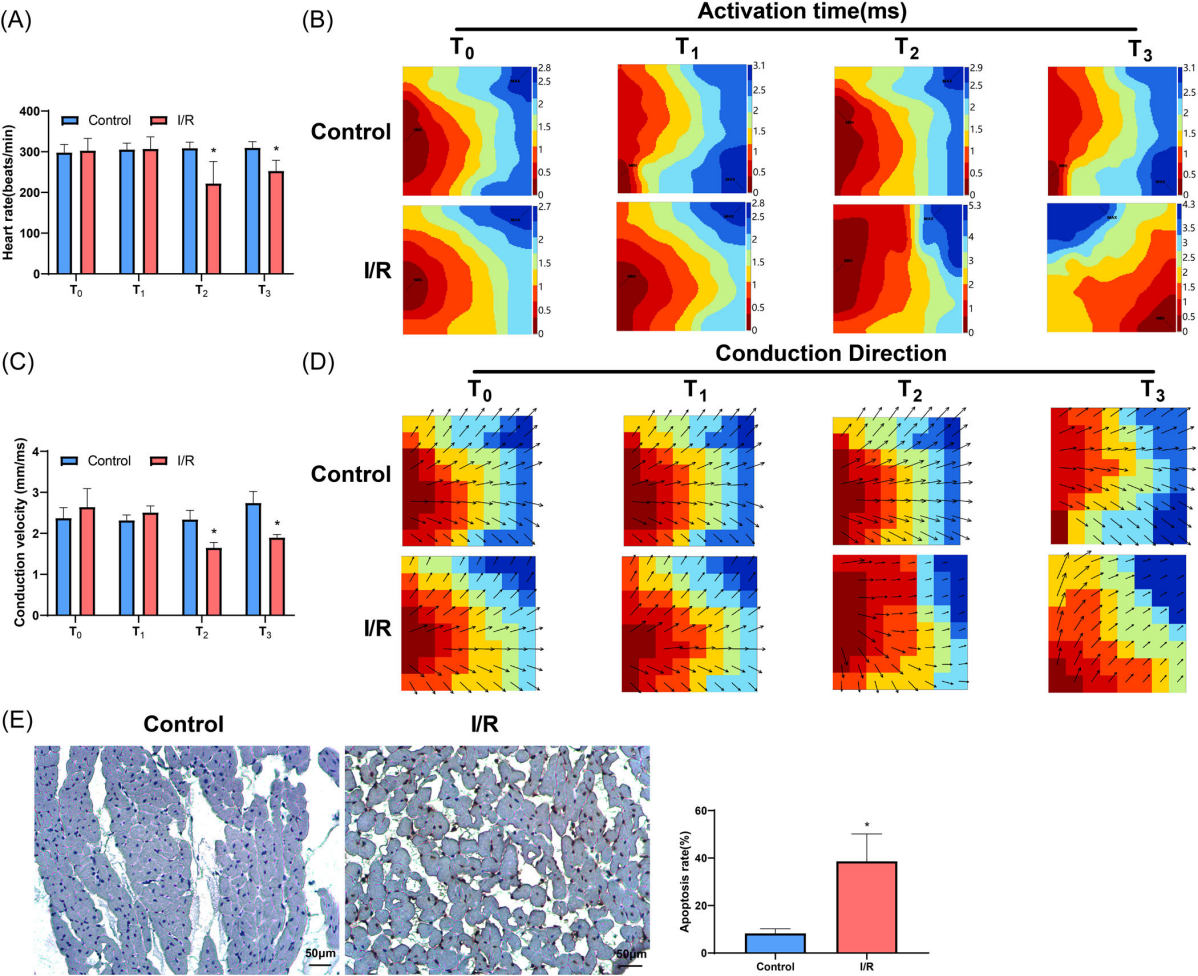
RA and cardiomyocyte apoptosis in the isolated Langendorff‐perfused rat heart model. After the development of the isolated Langendorff‐perfused heart model in rats, compared with the control group, the heart rate of rats at T_2_ and T_3_ in the I/R group was reduced (A); the MappingLab matrix electrophysiological mapping system showed increased conduction activation time (B), decreased conduction velocity (C), and changed conduction direction (D) at T_2_ and T_3_ in the left ventricle of the I/R group; myocardial tissues in the left ventricle were collected immediately after reperfusion, and TUNEL staining showed the apoptotic rate in myocardial tissues was obviously elevated in the I/R group (E). *n* = 8, *p < .05 compared with the control group. I/R, ischemia/reperfusion; RA, reperfusion arrhythmia; TUNEL, terminal deoxynucleotidyl transferase‐mediated dUTP nick‐end labeling.

First, we established an isolated Langendorff‐perfused rat heart model. None of the eight rats in the control group developed RA; the time for restoration of spontaneous heartbeat of the eight rats in the I/R group was 13.75 ± 7.78 s. In the I/R group, PVBs occurred in four rats (50%), and VF occurred in three rats (37.5%) including a rat with persistent VF at the end of the experiment. The score of RA in the I/R group was 2 ± 1.93, which was higher than that of the control group (0). Moreover, the heart rate at T_2_ and T_3_ was signally reduced in the I/R group compared with that in the control group (Figure 2A, *p* < .05). The conduction activation time, conduction velocity, and conduction direction of the left ventricle were tested by the MappingLab matrix electrophysiological mapping system. In the I/R group, the conduction activation time at T_2_ and T_3_ was increased (Figure 2B) and the conduction velocity at T_2_ and T_3_ was decreased in the I/R group (Figure 2C, *p* < .05), compared with those in the control group. The conduction direction at T_2_ and T_3_ was changed in the I/R group (Figure 2D). Myocardial tissues in the left ventricular were collected immediately after reperfusion, and TUNEL staining reflected that the apoptotic rate of cardiomyocytes in the I/R group was significantly higher than that in the control group (Figure 2E, *p* < .05). These results indicated that the isolated Langendorff‐perfused heart model was successfully established and induced RA and cardiomyocyte apoptosis.

### 3.2 Cx43 and STIP1 expression was decreased in the isolated Langendorff‐perfused rat heart model

**Figure 3:**
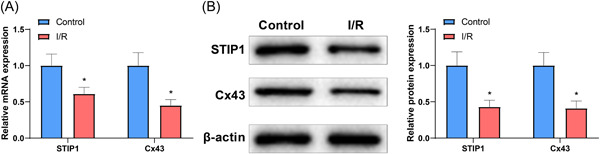
Low expression of Cx43 and STIP1 in the isolated Langendorff‐perfused rat heart model. Compared with the control group, qRT‐PCR (A) and western blotting (B) showed that the expression of Cx43 and STIP1 was reduced in myocardial tissues in the I/R group. *n* = 8, *p < .05 compared with the control group. Cx43, connexin 43; I/R, ischemia/reperfusion; qRT‐PCR, quantitative reverse transcription‐polymerase chain reaction; STIP1, stress‐induced phosphoprotein 1.

Next, we performed qRT‐PCR and western blotting to detect the expression of Cx43 and STIP1 in RA, and the results showed that the expression of Cx43 and STIP1 in the I/R group was dramatically reduced compared with that in the control group (Figure 3A,B, *p* < .05). The above data suggested obviously decreased expression of Cx43 and STIP1 in the isolated Langendorff‐perfused rat heart model of RA.

### 3.3 Cx43 and STIP1 were downregulated in H9C2 cellular model of H/R

**Figure 4:**
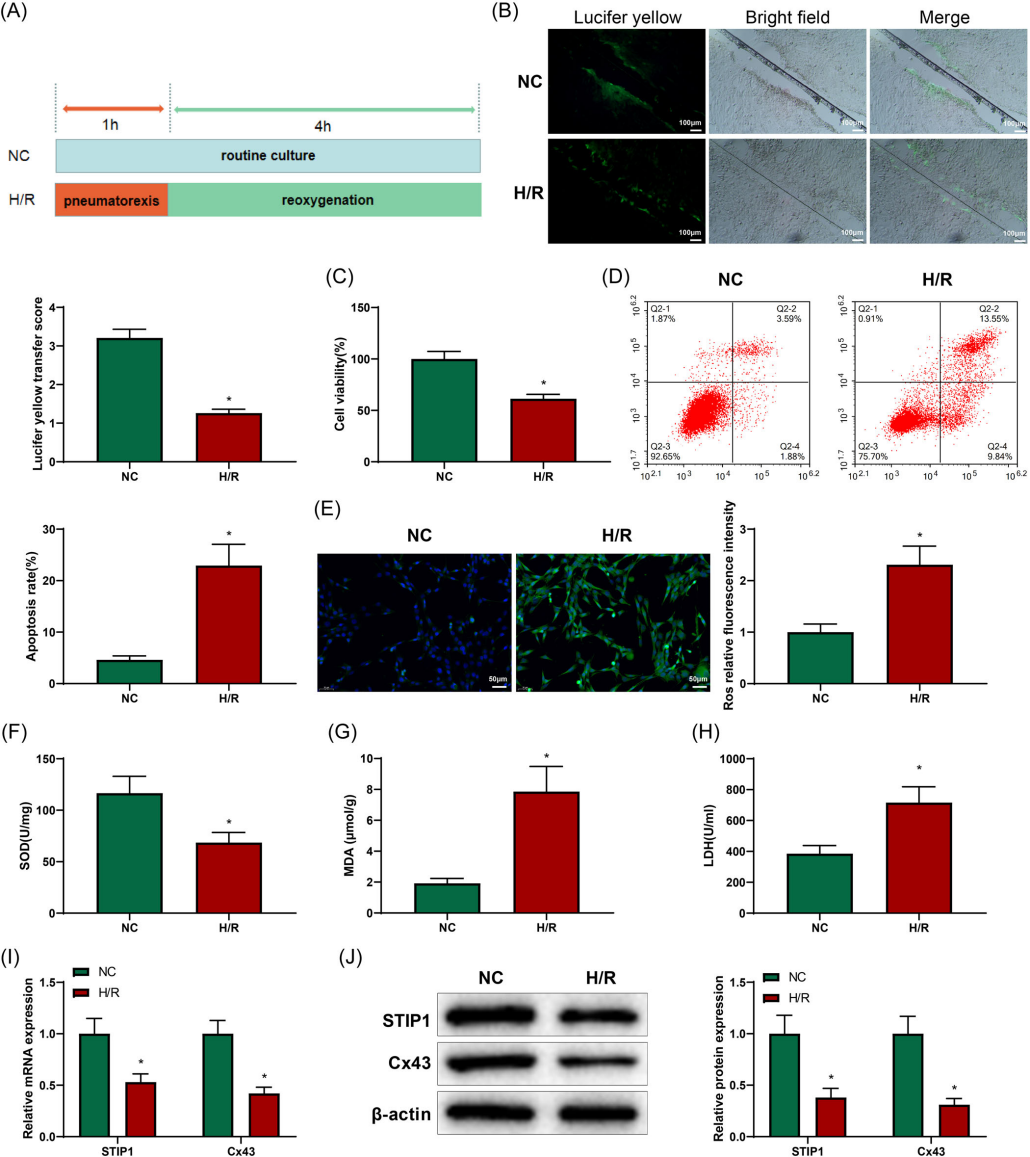
Downregulation of Cx43 and STIP1 in the H/R cellular model. The H9C2 cellular model of H/R was constructed (A). Compared with the NC group, SLDT assay showed decreased intercellular gap junctions in the H/R group (B); CCK‐8 assay revealed that H9C2 cell viability was decreased in the H/R group (C); flow cytometry revealed increased H9C2 cell apoptosis in the H/R group (D); the elevated level of ROS was detected by DCFH‐DA in the H/R group (E); the declined level of SOD, and increased levels of MDA and LDH were tested by ELISA in the H/R group (F–H); qRT‐PCR (I) and western blotting (J) showed that the expression of Cx43 and STIP1 was downregulated in cardiomyocytes in the H/R group. Three independent experiments were performed, *p < .05 compared with the NC group. CCK‐8, cell counting kit 8; Cx43, connexin 43; DCFH‐DA, 2′,7′‐dichlorodihydrofluorescein; ELISA, enzyme‐linked immunosorbent assay; H/R, hypoxia/reoxygenation; LDH, lactic dehydrogenase; MDA, malondialdehyde; NC, negative control; qRT‐PCR, quantitative reverse transcription‐polymerase chain reaction; ROS, reactive oxygen species; SLDT, scrape‐loading and dye transfer; SOD, superoxide dismutase; STIP1, stress‐induced phosphoprotein 1.

To further detect the expression of Cx43 and STIP1 in the cellular model, we induced the H/R cell model in H9C2 cells (Figure 4A). Reportedly, the pretreatment of Gap19 (200 μM; 30 min) could significantly inhibit Cx43 hemichannel activity without disturbing GJs.^9^ Therefore, we pretreated H9C2 cells with Gap19 (200 μM) for 30 min to eliminate the interference of hemichannel activation in cells. The intensity of intercellular GJs in each group was measured by SLDT, and the results revealed that the score of fluorescence transmission in the H/R group was decreased compared with that in the NC group (Figure 4B), suggesting a decrease in intercellular GJs. The results of CCK‐8 and flow cytometry indicated that the H/R group had lower cell viability and higher apoptotic rate than the NC group (Figure 4C,D, *p* < .05). DCFH‐DA results demonstrated enhanced fluorescence intensity in the H/R group compared with that in the NC group (Figure 4E, *p* < .05), indicating an increase in ROS level in the H/R group. ELISA results showed decreased SOD content and elevated MDA and LDH levels in the H/R group compared with those in the NC group (Figure 4F–H, *p* < .05). Consistent with the results obtained from the animal model, the expression of Cx43 and STIP1 in the H/R group was markedly reduced compared with that in the NC group (Figure 4I,J, *p* < .05). Taken together, Cx43 and STIP1 were significantly reduced in H/R‐induced cardiomyocytes.

### 3.4 Cx43 directly bound to HSP70 and HSP90

**Figure 5:**
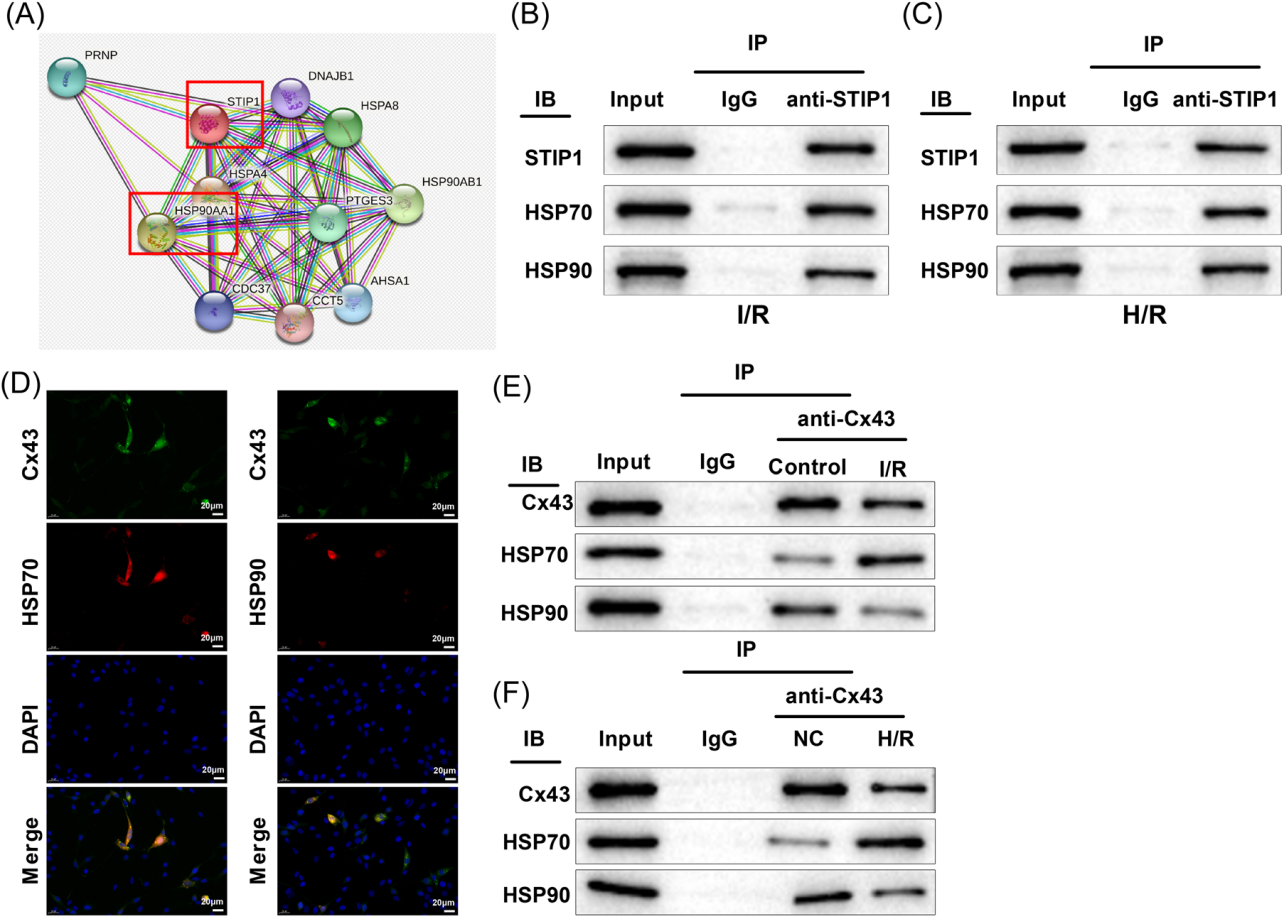
Binding of Cx43 to HSP70 and HSP90. The string database predicted a potential interaction between STIP1 and HSP90 (A). Co‐IP assay showed that STIP1 could directly bind to HSP90 and HSP70 in myocardial tissues of the I/R group (B) and H9C2 cells of the H/R group (C). Fluorescence co‐localization assay revealed that Cx43‐HSP70 and Cx43‐HSP90 existed in complex forms (D). Co‐IP assay displayed that Cx43 bound to HSP90 and HSP70 in rat myocardial tissues, where compared with the control group, HSP70 expression was increased and HSP90 expression was decreased in the immune complex pulled down by the Cx43 antibody in the I/R group (E). Co‐IP assay displayed that Cx43 bound to HSP90 and HSP70 in H9C2 cells, where compared with the NC group, HSP70 expression was increased and HSP90 expression was decreased in the immune complex pulled down by the Cx43 antibody in the H/R group (F). Each experiment was independently repeated three times. Co‐IP, co‐immunoprecipitation; Cx43, connexin 43; H/R, hypoxia reoxygenation; I/R, ischemia reperfusion; NC, negative control; STIP1, stress‐induced phosphoprotein 1.

To clarify the specific mechanisms of Cx43 and STIP1 in RA, we used the string database (https://cn.string-db.org/) and found a possible interaction between STIP1 and HSP90 (Figure 5A). Co‐IP results suggested that STIP1 directly bound to HSP70 and HSP90 in the I/R and H/R groups (Figure 5B,C).

In addition, fluorescence co‐localization assay revealed that Cx43 colocalized with HSP90 and HSP70 in the cytoplasm (Figure 5D), indicating that Cx43‐HSP70 and Cx43‐HSP90 could exist in complex forms in cells. Co‐IP assay results showed that HSP70 and HSP90 proteins were pulled down by the anti‐Cx43 antibody. Furthermore, HSP70 expression was increased and HSP90 expression was decreased in the immune complex pulled down by the Cx43 antibody in the I/R and H/R groups, compared with the control and NC groups (Figure 5E,F). Overall, Cx43, a client protein, could bind to HSP70 and HSP90, and the Cx43‐HSP90 complex decreased but the Cx43‐HSP70 complex increased in I/R‐induced myocardial tissues and H/R‐treated cardiomyocytes.

### 3.5 HSP90 inhibited Cx43 ubiquitination

**Figure 6:**
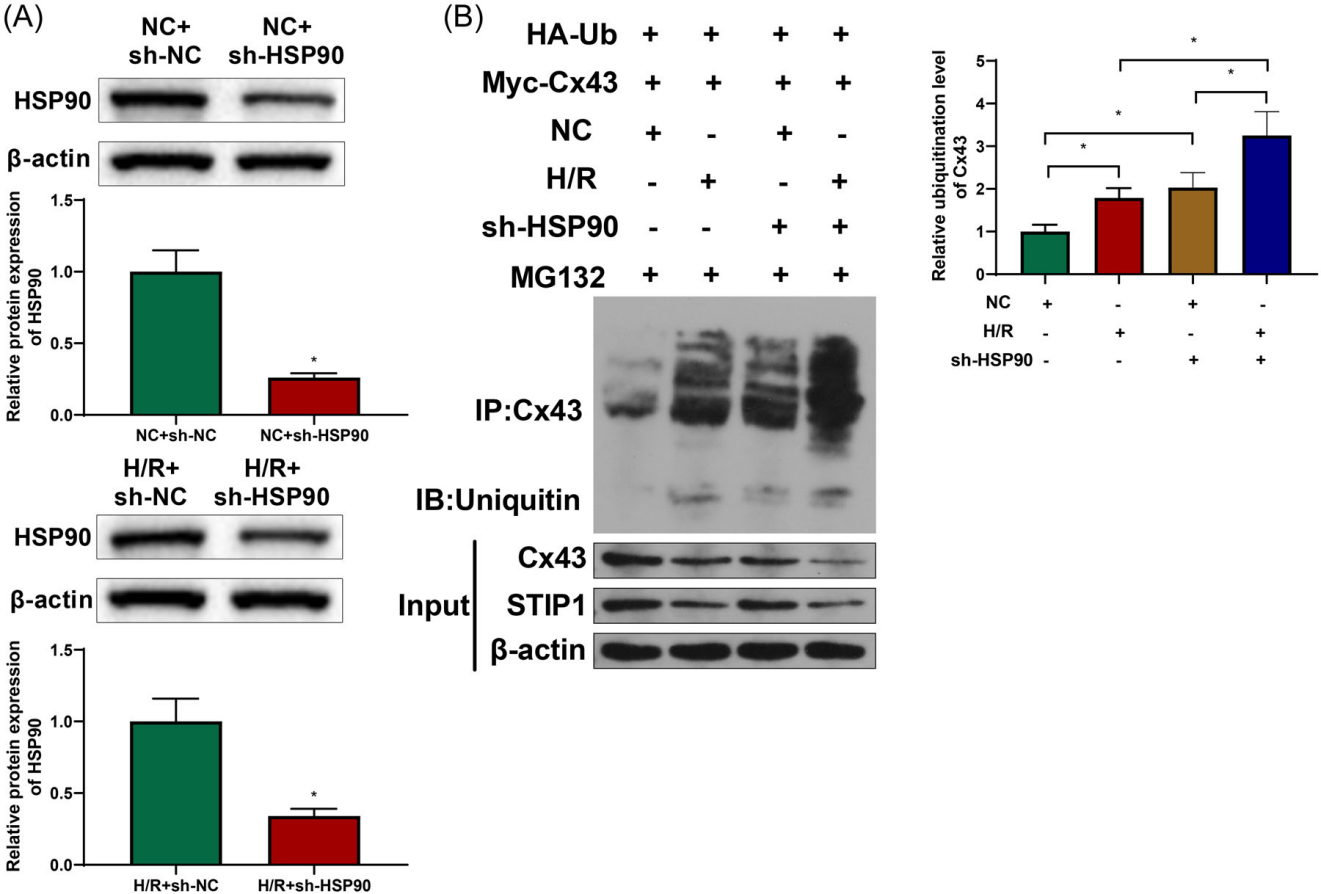
Inhibitory effect of HSP90 on Cx43 ubiquitination. Western blotting showed that sh‐HSP90 transfection inhibited the expression of HSP90 (A). Co‐IP assay illustrated that the ubiquitination level of Cx43 in the H/R group was higher than that in the NC group and that sh‐HSP90 transfection further enhanced Cx43 ubiquitination (B). Each assay was run in triplicate, *p < .05 compared with the NC + sh‐NC, H/R + sh‐NC, NC, H/R, or NC + sh‐HSP90 group. Co‐IP, co‐immunoprecipitation; H/R, hypoxia reoxygenation; NC, negative control; sh‐HSP90, HSP90 knockdown vector.

Based on the above results, we hypothesized that Cx43 was recruited as a client protein by HSP70 and assembled into a complex with HSP70; with the help of STIP1, Cx43 was transferred to HSP90, which further promoted and activated Cx43 to increase its stability and inhibit Cx43 ubiquitination, ultimately participating in the progression of RA. Toward this end, we transfected sh‐HSP90 into cardiomyocytes in the NC and H/R groups, and 3 h later, western blotting found that the transfection of sh‐HSP90 significantly inhibited the expression of HSP90 (Figure 6A, *p* < .05). Next, Co‐IP assay was used to detect the ubiquitination level of Cx43 in each group, and results displayed that the Cx43 ubiquitination level in the H/R group was clearly higher than that in the NC group, and the transfection of sh‐HSP90 further increased Cx43 ubiquitination (Figure 6B, *p* < .05). These results demonstrated that HSP90 could repress Cx43 ubiquitination.

### 3.6 STIP1 improved intercellular communication and reduced oxidative stress in H/R‐treated H9C2 cells by increasing Cx43 protein expression

**Figure 7:**
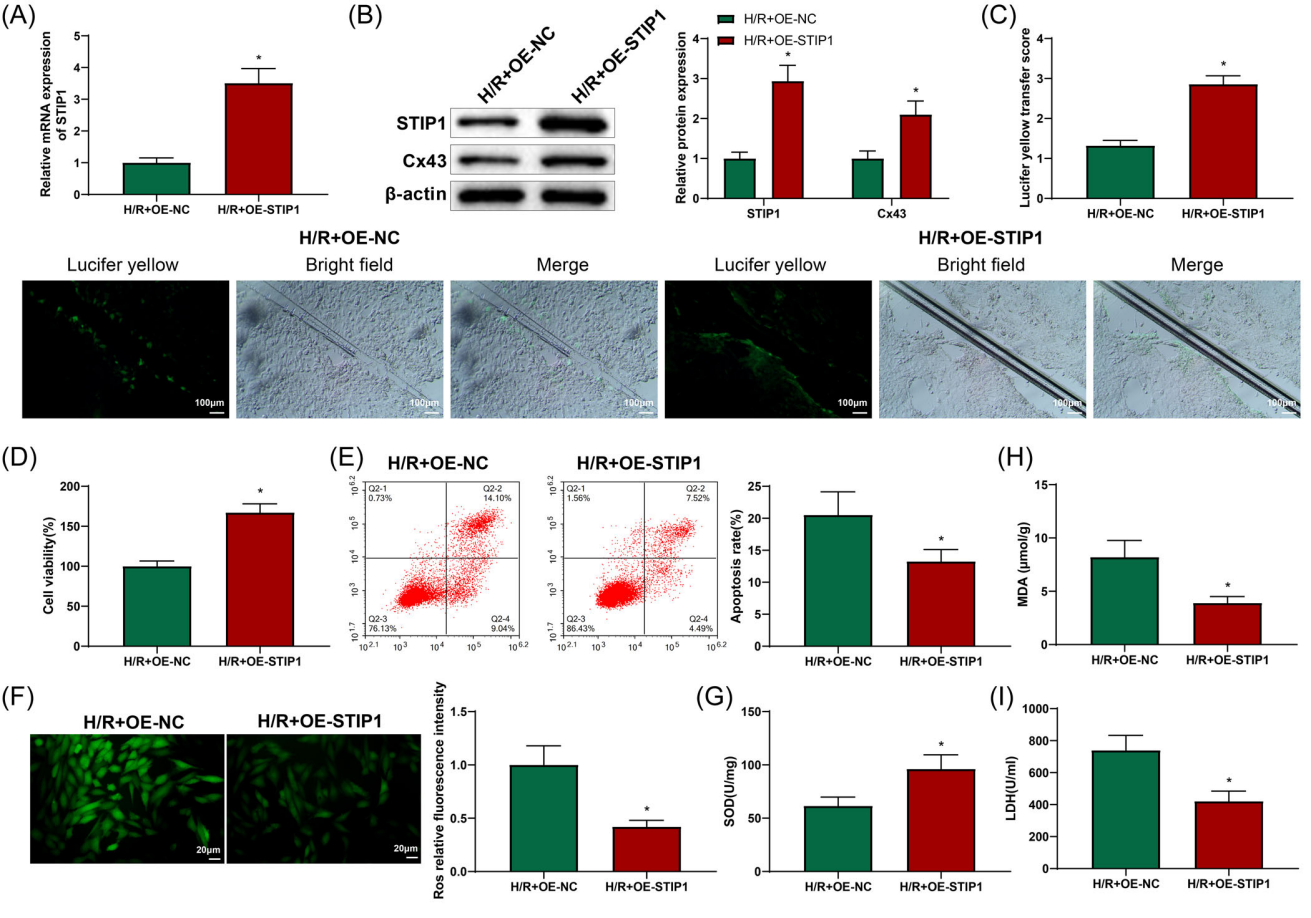
Effects of STIP1 on intercellular communication and oxidative stress in H/R‐treated H9C2 cells. Compared with the H/R + OE‐NC group, qRT‐PCR showed the successful transfection of OE‐STIP1 (A). After overexpression of STIP1, western blotting showed that the protein expression of STIP1 and Cx43 was elevated (B); SLDT assay showed enhanced intercellular gap junctions (C); CCK‐8 assay showed increased H9C2 cell viability (D); flow cytometry revealed decreased H9C2 cell apoptosis (E); DCFH‐DA assay exhibited the reduced level of ROS (F); ELISA displayed the upregulated level of SOD and downregulated levels of MDA and LDH (G–I). Three independent experiments were performed. *p < .05 compared with the H/R + OE‐NC group. CCK‐8, cell counting kit 8; Cx43, connexin 43; DCFH‐DA, 2′,7′‐dichlorodihydrofluorescein; ELISA, enzyme‐linked immunosorbent assay; H/R, hypoxia/reoxygenation; LDH, lactic dehydrogenase; MDA, malondialdehyde; NC, negative control; oe, overexpression; qRT‐PCR, quantitative reverse transcription‐polymerase chain reaction; ROS, reactive oxygen species; SLDT, scrape‐loading and dye transfer; SOD, superoxide dismutase; STIP1, stress‐induced phosphoprotein 1.

H9C2 cells were transduced with OE‐STIP1 before exposure to H/R to further determine the regulation of Cx43 by STIP1. qRT‐PCR and western blotting revealed that the H/R + OE‐STIP1 group had increased STIP1 mRNA and protein expression and elevated Cx43 protein expression versus H/R + OE‐NC group (Figure 7A,B, *p* < .05). Depicted by SLDT assay, the score of fluorescence transmission was observably enhanced in the H/R + OE‐STIP1 group than in the H/R + OE‐NC group (Figure 7C, *p* < .05), suggesting an increase in intercellular GJs. CCK‐8 and flow cytometry results displayed that cell viability was elevated but cell apoptosis was reduced in the H/R + OE‐STIP1 group than in the H/R + OE‐NC group (Figure 7D,E, *p* < .05). DCFH‐DA results suggested weakened fluorescence intensity in the H/R + OE‐STIP1 group compared with that in the H/R + OE‐NC group (Figure 7F, *p* < .05). As expected, ELISA results reflected increased SOD content and decreased MDA and LDH levels in the H/R + OE‐STIP1 group compared to those in the H/R + OE‐NC group (Figure 7G–I, *p* < .05). In summary, STIP1 facilitated Cx43 protein expression to improve intercellular communication, promote cell viability, and reduce oxidative stress and cell apoptosis in H/R‐treated H9C2 cells.

## 4 DISCUSSION

In our study, we first established an isolated Langendorff‐perfused rat heart model and an H/R injury model in H9C2 cells where Cx43 and STIP1 were detected to be downregulated in the myocardial tissues and cells. Furthermore, with STIP1 as a co‐chaperone, Cx43 preferentially bound to HSP90, which further inhibited Cx43 ubiquitination and increased Cx43 expression.

GJ trafficking plays an important role in onset and progression of cardiac arrhythmia.^21^ As the most common component of GJ channels, Cx43 was downregulated in RA,^22^ which was consistent with our results. In I/R‐damaged hearts, downregulation and redistribution of Cx43 caused arrhythmia and the expansion of myocardial infarction area.^23^ These data suggest that Cx43 plays an important role in the occurrence and development of RA. Cx43 hemichannel activation during diastolic release in ventricular cardiomyocytes enhanced Ca^2+^ dynamics, and in heart failure, increased hemichannel activity promoted electrical excitability, which may be a reasonable explanation of cardiac arrhythmia.^24^ As illustrated by Lillo et al.,^25^ the blockade of Cx43 hemichannel activation or the decrease in Cx43 levels curtailed the aberrant increase in membrane permeability, plasma membrane depolarization, and isoproterenol‐evoked electrical activity in mutant X‐linked dystrophin (*Dmd* ^*mdx*^) mouse cardiomyocytes. On the contrary, upon the stimulation of cardiac stress, the opening of Cx43 hemichannel facilitated the release of Ca^2+^, depolarization, and triggered activities in ventricular cardiomyocytes from patients with heart failure.^26^ These findings showed that Cx43 hemichannels could serve as a novel therapeutic target that can prevent cardiac arrhythmias and heart dysfunction.

In addition to decreased Cx43 expression, our functional experiments displayed that STIP1 was downregulated in the animal and cellular models. Another study indicated that STIP1 treatment prevented ischemia‐mediated cell apoptosis, indicating the protective role of STIP1 against ischemia‐induced injury.^27^ However, the mechanisms of STIP1 in RA remain poorly understood. Moreover, the string database manifested that STIP1 could bind to HSP90, and Co‐IP results further confirmed that STIP1 directly bound to HSP90 and HSP70. A previous study demonstrated that STIP1 could help mature clients by physically linking to HSP70 and HSP90, allowing client transfer from HSP70 to HSP90.^28^ HSP70‐HSP90 machinery has long been reported to direct their substrates toward ubiquitination degradation.^29^ HSP90 stabilizes the conformation of a substrate protein by forming a multi‐molecular chaperone complex with the protein, and HSP90 deficiency promotes ubiquitination and degradation of its substrate protein.^30^ In addition, STIP1 homology and U‐box‐containing protein 1, an E3 ubiquitin ligase, was reported to promote the ubiquitination and degradation of a downstream gene.^31^ Previous data suggested that Cx43 was regulated by several E3 ubiquitin ligases.^32^ Wassim A. Basheer et al. found that Cx43 ubiquitination was associated with the occurrence of RA.^33^

The ubiquitin system is complex, multifaceted, and crucial for regulating a large number of cellular processes, and ubiquitination is the key to the dynamic regulation of programmed cell death.^34^ Follow‐up experiments revealed that Cx43 was colocalized with HSP90 and HSP70 in the cytoplasm, indicating that Cx43‐HSP70 and Cx43‐HSP90 existed in complex forms in cells. In H/R‐injured cardiomyocytes, the Cx43‐HSP70 complex was increased and the Cx43‐HSP90 complex was decreased. After the transfection of sh‐HSP90, Cx43 ubiquitination was further promoted in H/R‐injured cardiomyocytes. Overexpression of STIP1 in cardiomyocytes substantially facilitated intercellular communication, prevented oxidative stress injury and apoptosis, and promoted viability after H/R stimulation. Therefore, we speculated that Cx43, a client protein, was transferred to HSP90 with the help of STIP1, which further activated Cx43, inhibited Cx43 ubiquitination, and then participated in RA progression. However, this speculation needs to be further validated by more extensive experiments at animal and clinical levels in the future. Apart from Cx43 hemichannel activation and phosphorylation, our results provided a possible explanation for Cx43‐related RA.

## 5 CONCLUSION

To summarize, we found decreased Cx43 and STIP1 in both animal and cellular models of RA. Experiments validated that Cx43 and STIP1 could directly bind to HSP90 and HSP70, and STIP1 improved intercellular communication and reduced oxidative stress of H/R H9C2 cells by elevating Cx43 protein expression. Although interesting results have been obtained in our experimental studies, these findings have not been translated into clinical benefits. Therefore, more research is needed in the future to further characterize this molecular mechanism.

## AUTHOR CONTRIBUTIONS

**Li An, Hong Gao**: conceptualization (equal). **Hong Gao**: supervision (lead). **Li An, Hong Gao**: designed the experiments (equal). **Li An, Yi Zhong, Yanqiu Liu, Ying Cao, Jing Yi**: performed the experiments (equal). **Li An, Yi Zhong, Xiang Huang, Chunlei Wen, Rui Tong, Zhijun Pan, Xu Yan, Meiyan Liu, Shengzhao Wang, Hao Wu, Tingju Hu**: analyzed the data (equal). **Li An, Yanqiu Liu, Ying Cao**: provided critical materials (equal). **Rui Tong, Zhijun Pan, Xu Yan, Meiyan Liu**: writing—original draft (equal).

## CONFLICT OF INTEREST STATEMENT

The authors declare no conflicts of interest.

## Supporting information

Supporting information.

## Data Availability

The datasets used or analyzed during the current study are available from the corresponding author on reasonable request.
