# Supplementary material for: The potential roles of stress‐induced phosphoprotein 1 and connexin 43 in rats with reperfusion arrhythmia
Source: Immun Inflamm Dis. 2023 Oct 3;11(10):e852. doi: 10.1002/iid3.852 (PMC10546868; doi:10.1002/iid3.852)
Supplement: Supplementary file 1 — Supporting information. [file IID3-11-e852-s001.docx]

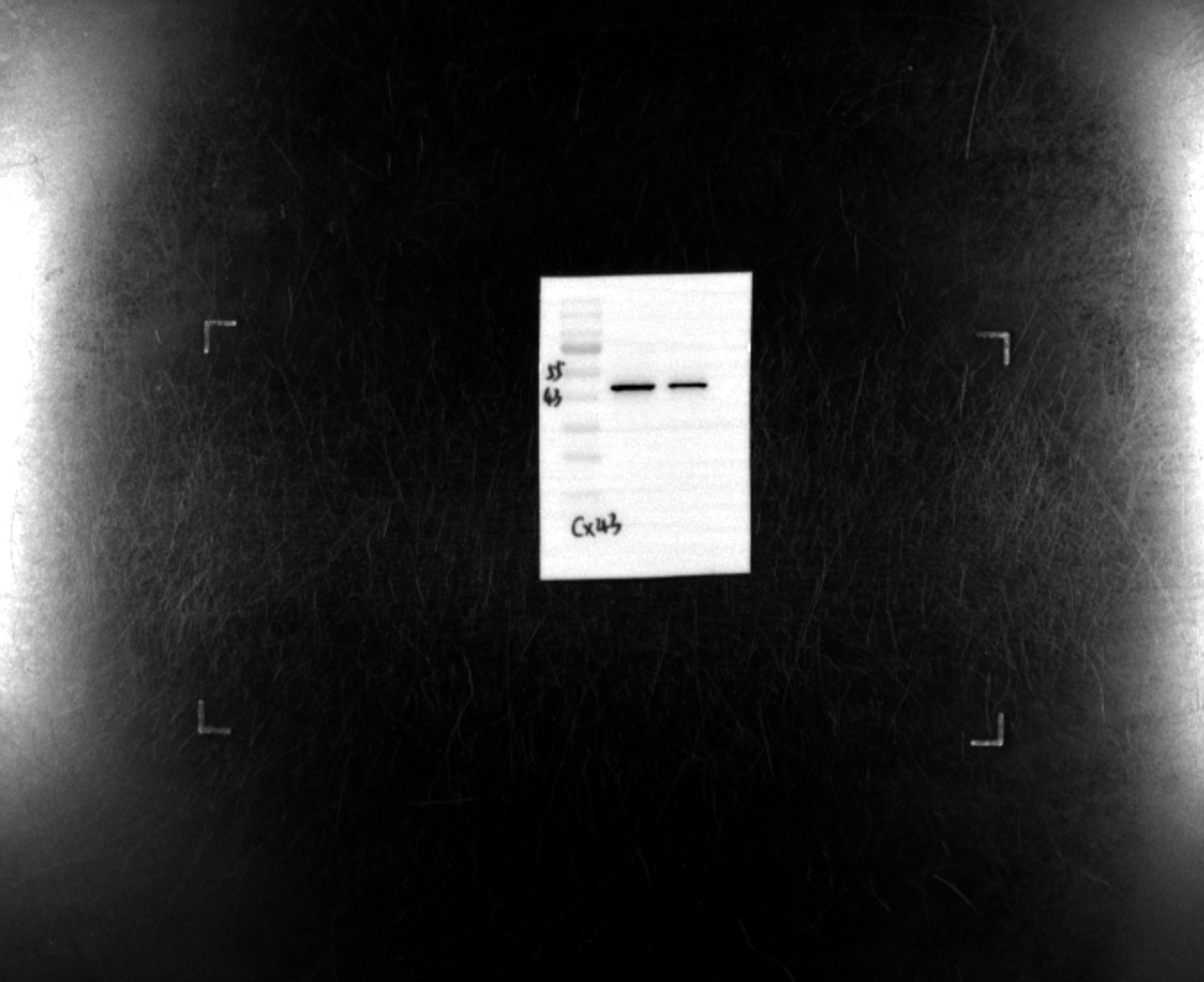


3B- CX43


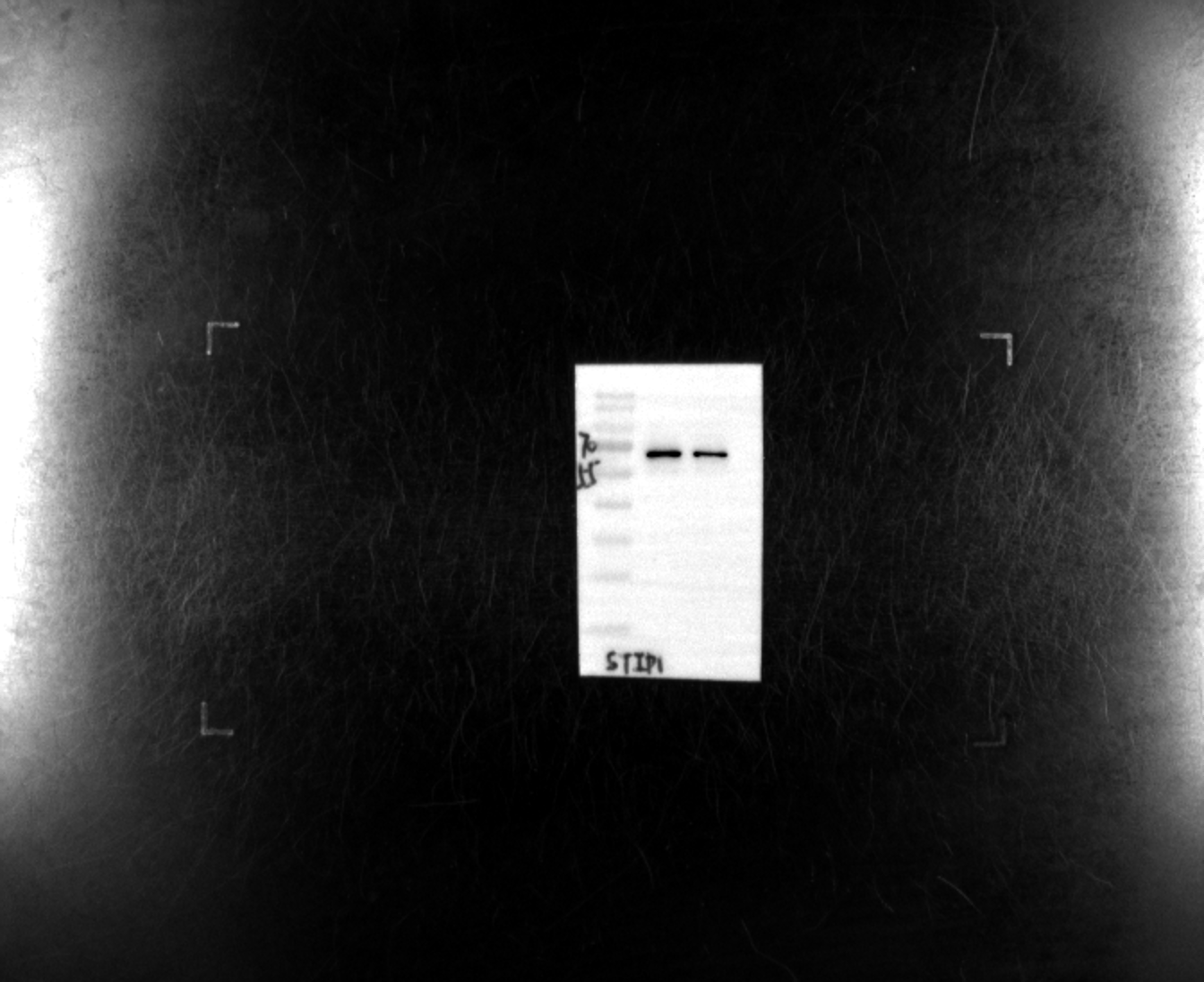


3B-STIP1


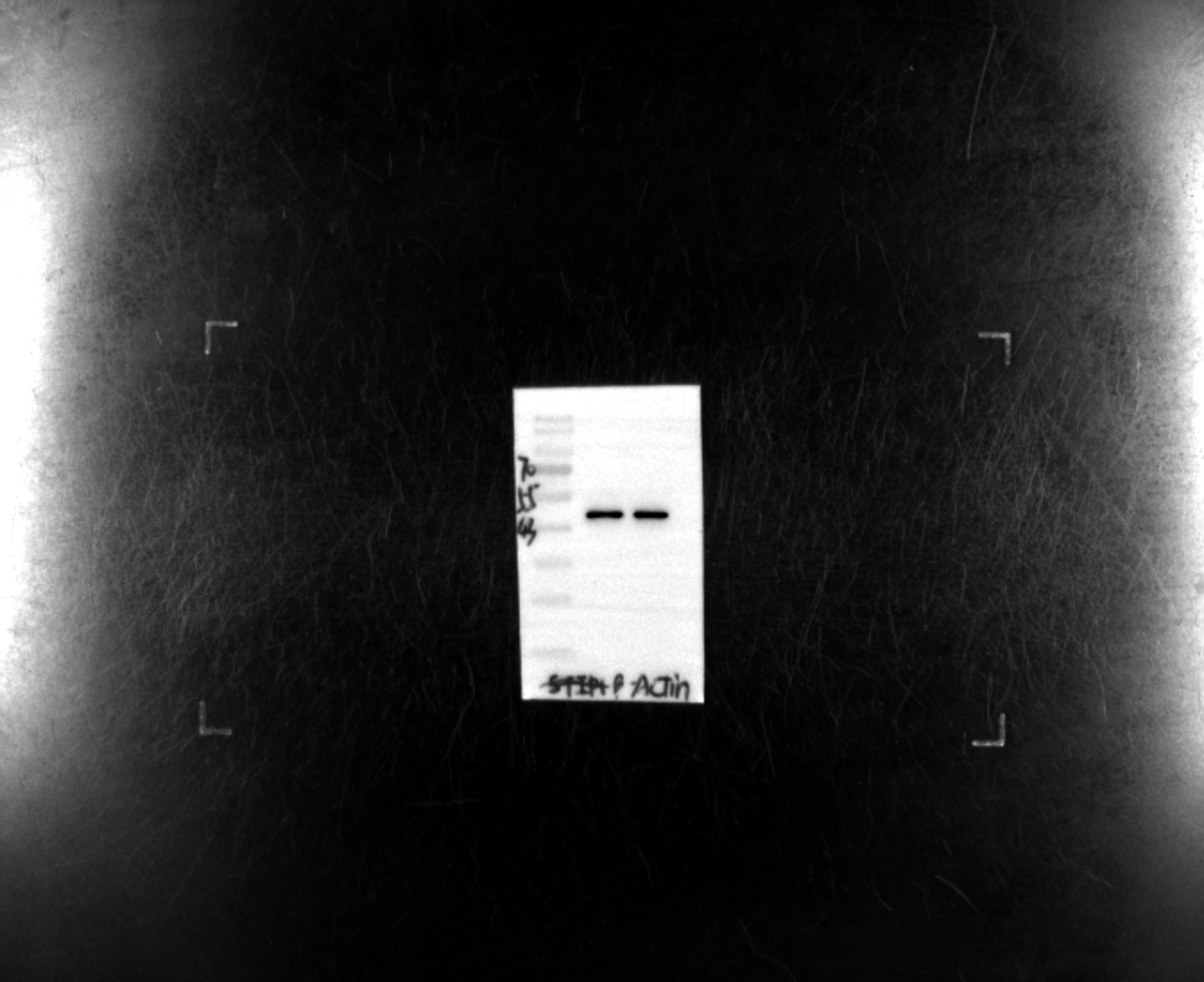


3B-β-ACTIN


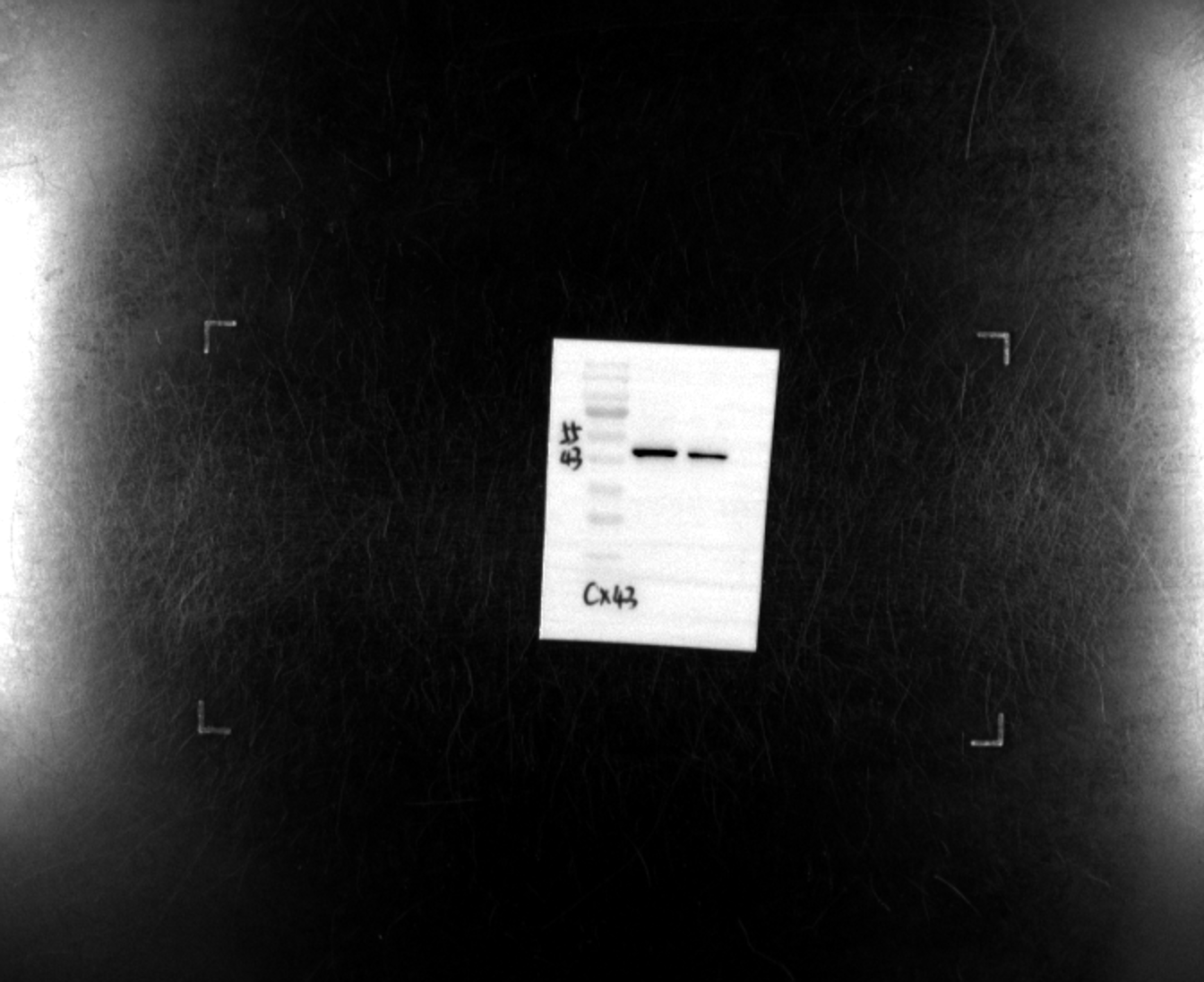


4J-CX43


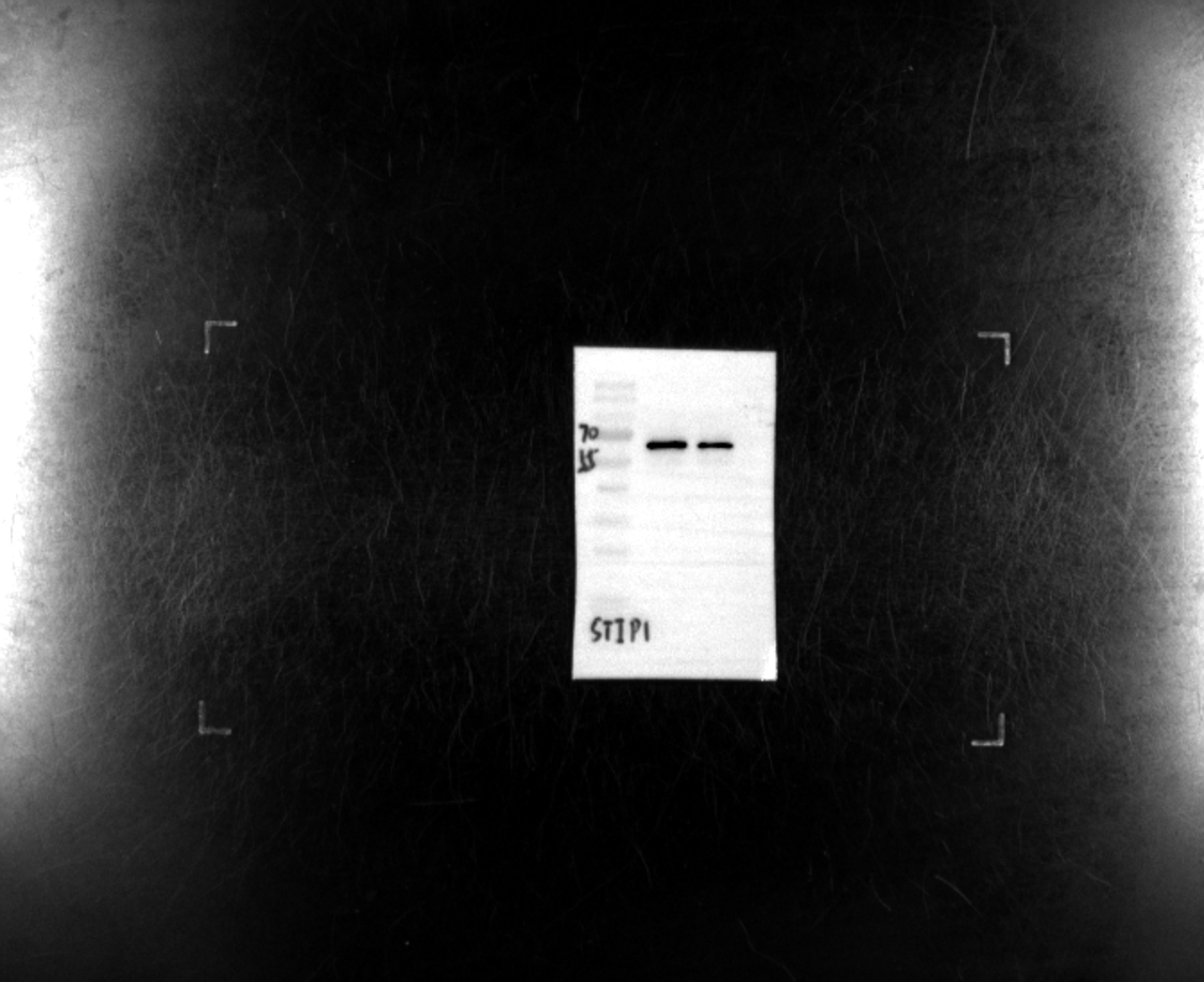


4J-STIP1


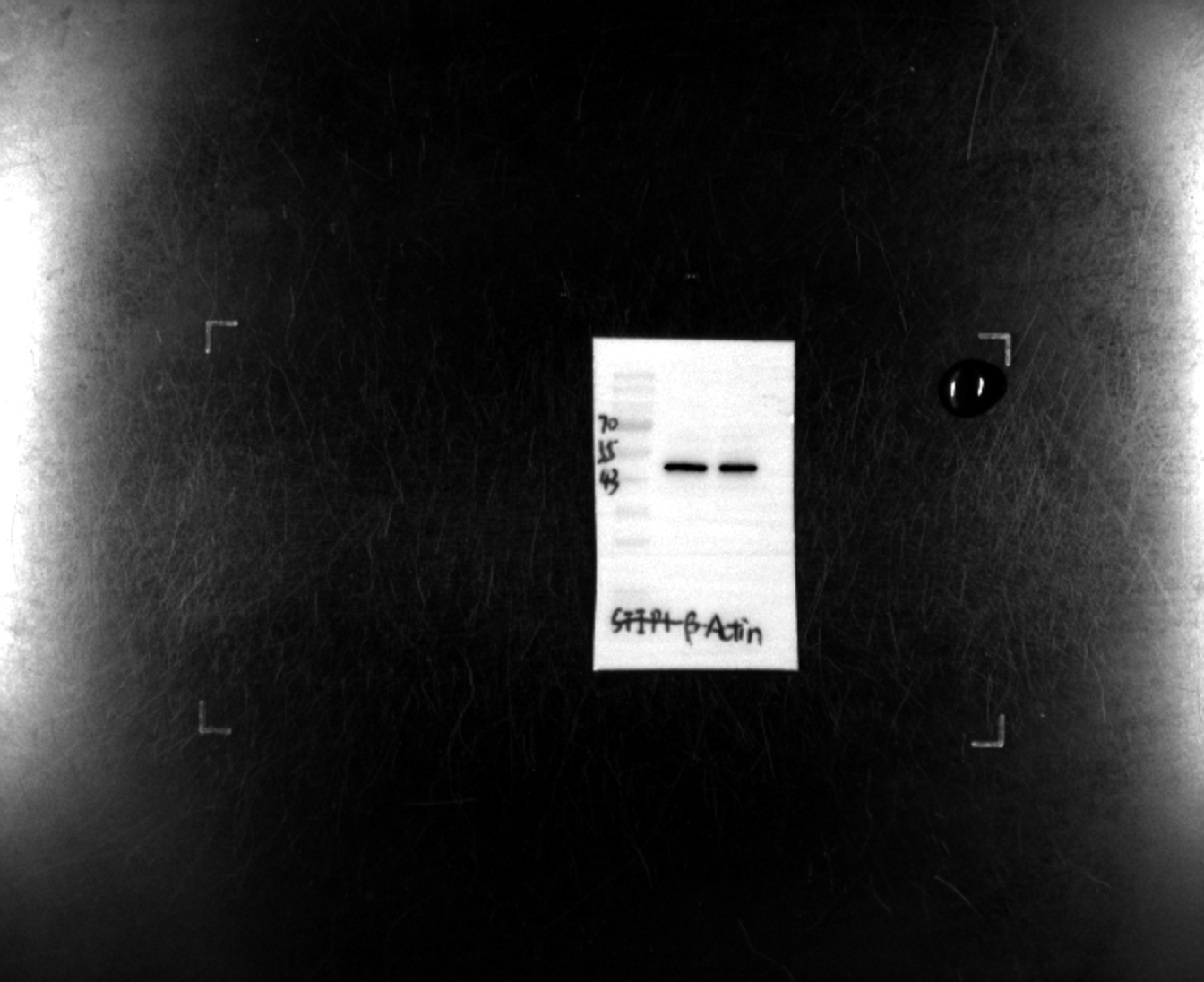


4J-β-ACTIN


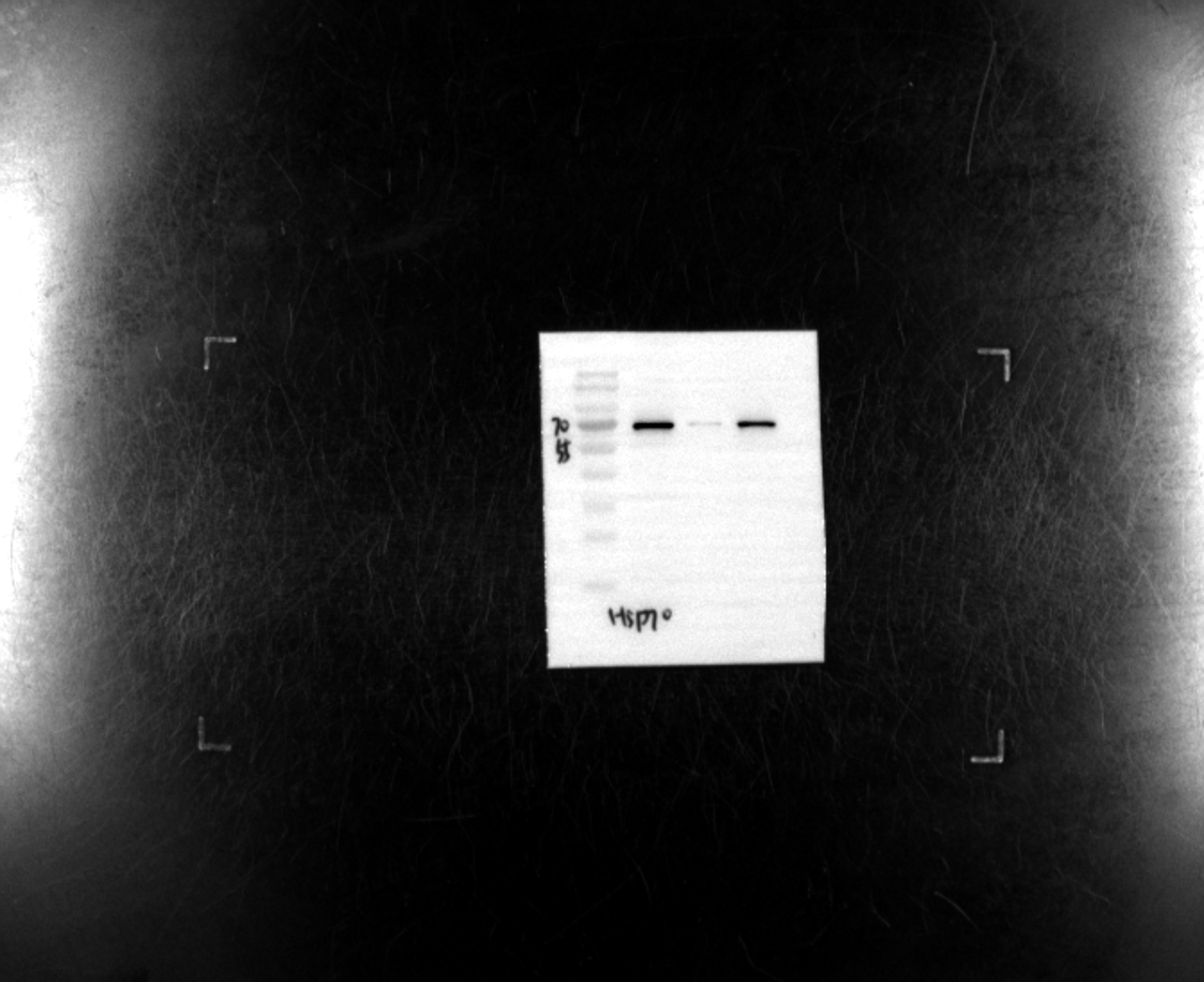


5B-HSP70


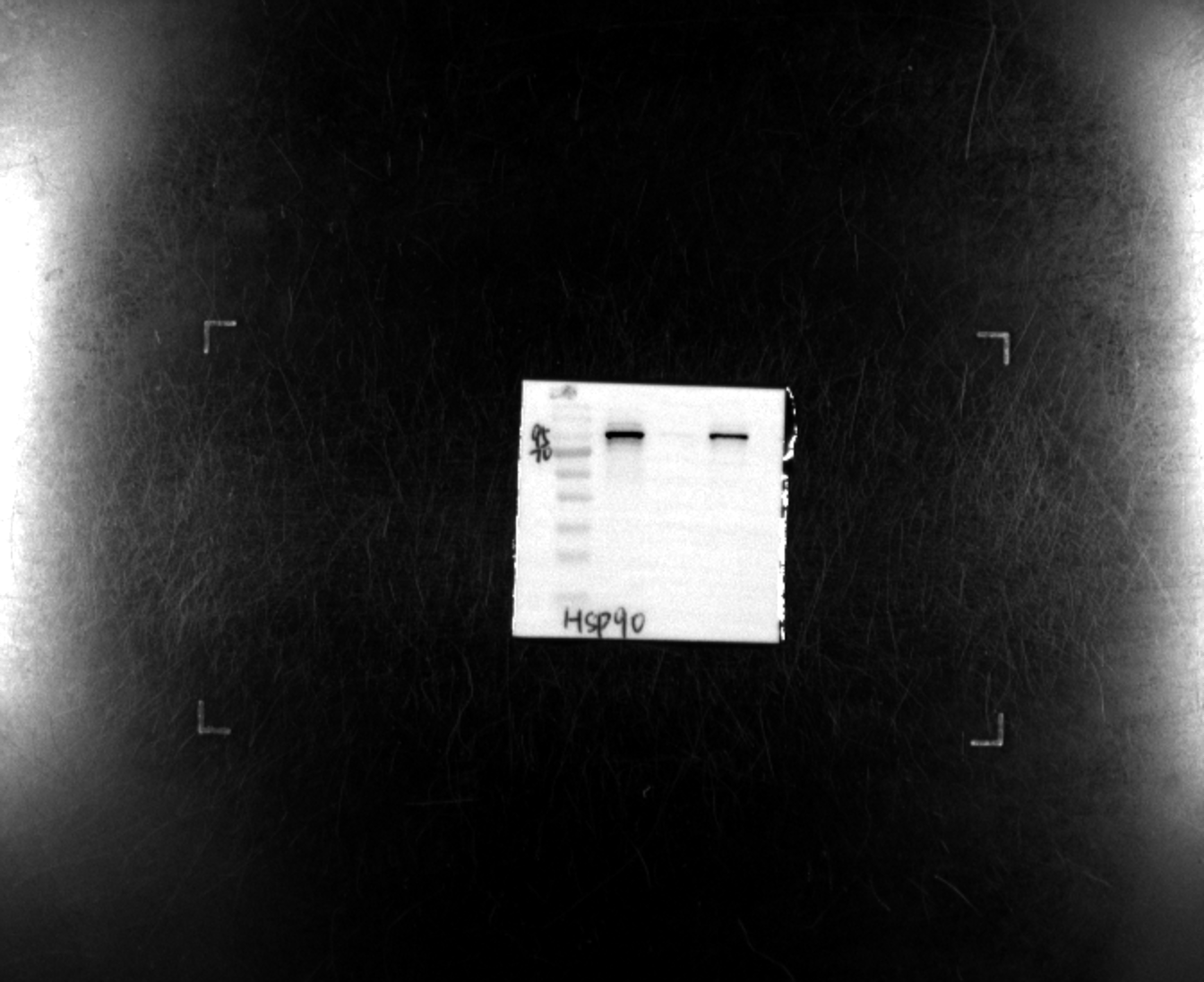


5B-HSP90


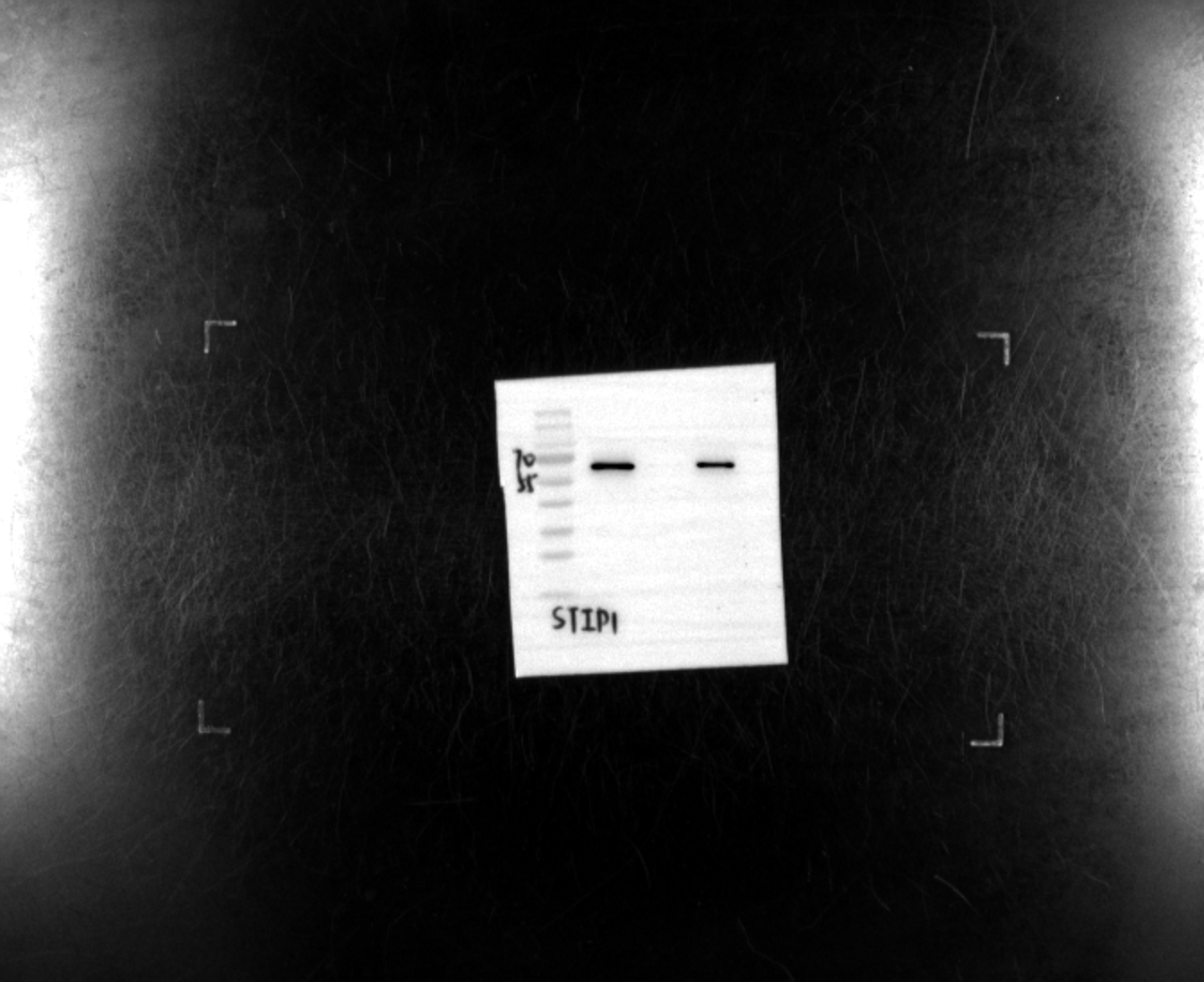


5B-STIP1


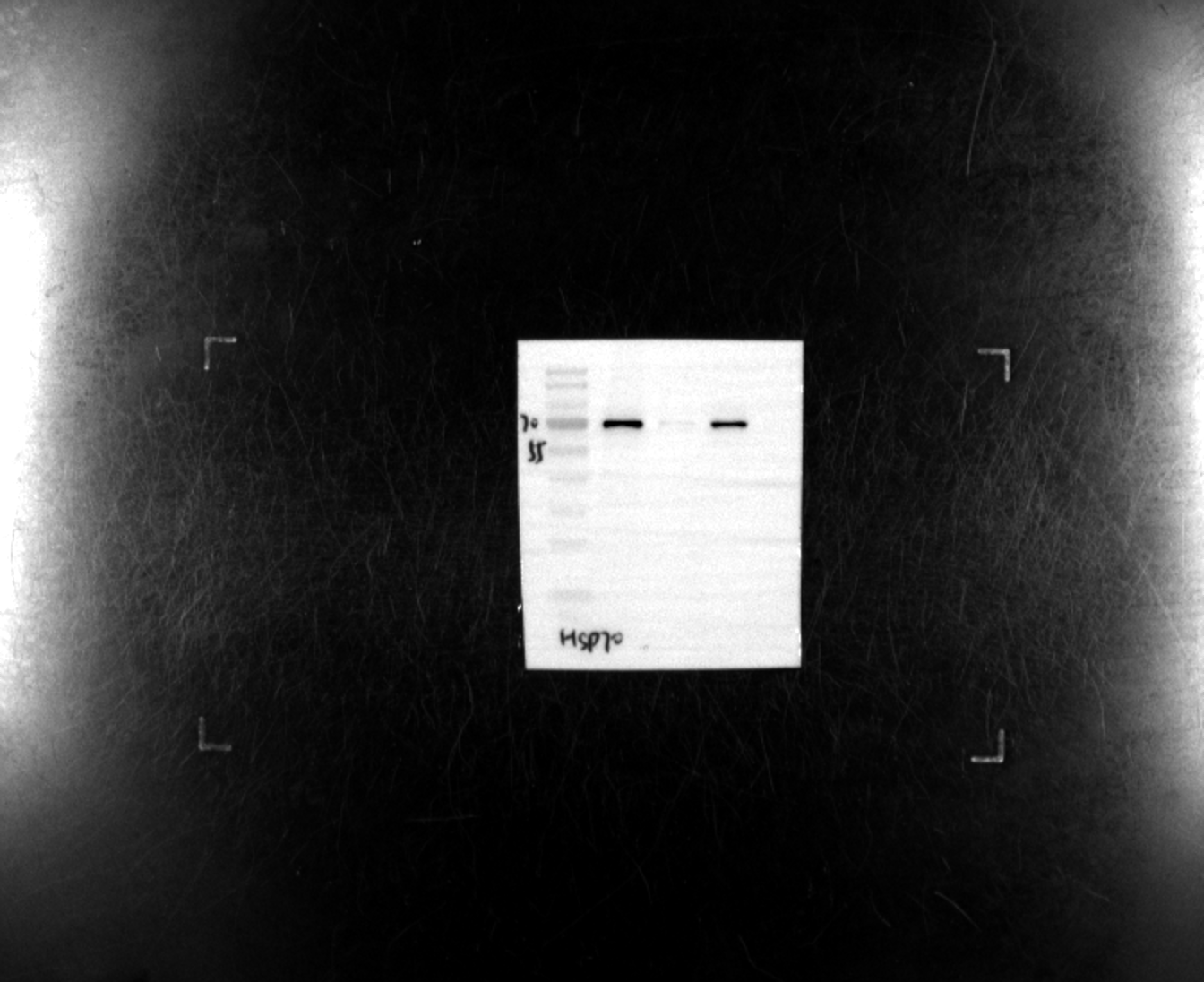


5C-HSP70


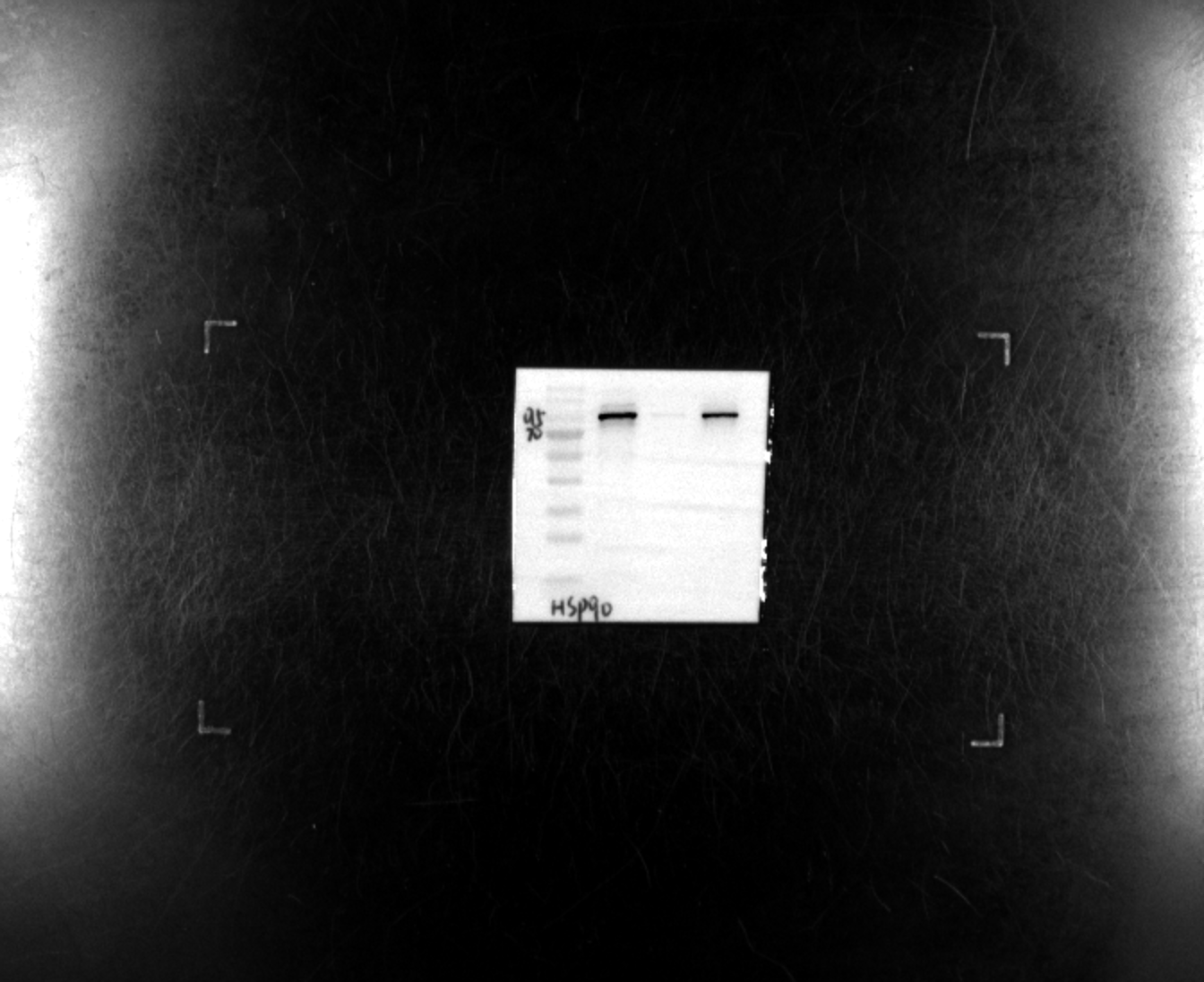


5C-HSP90


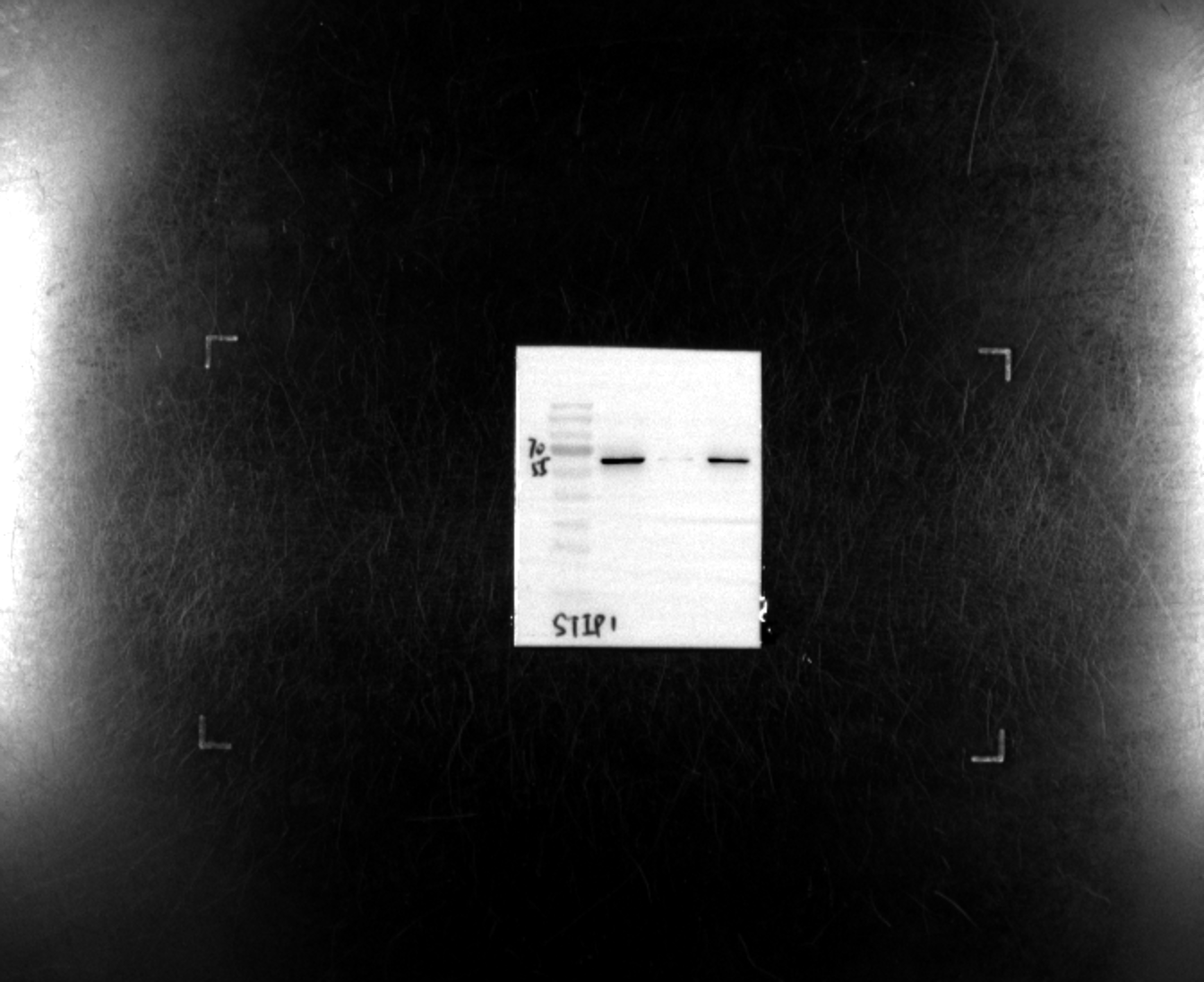


5C-STIP1


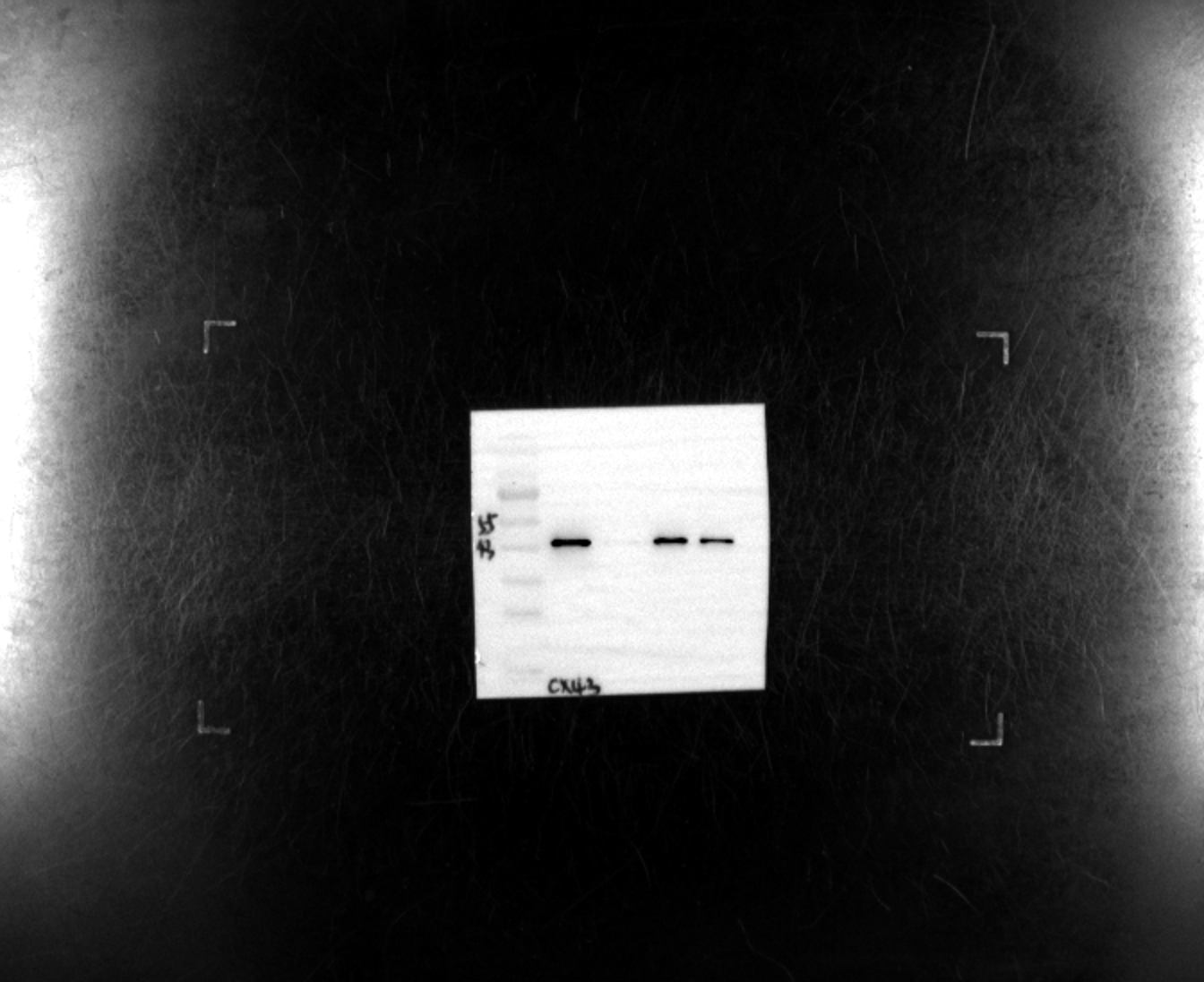


5E-CX43


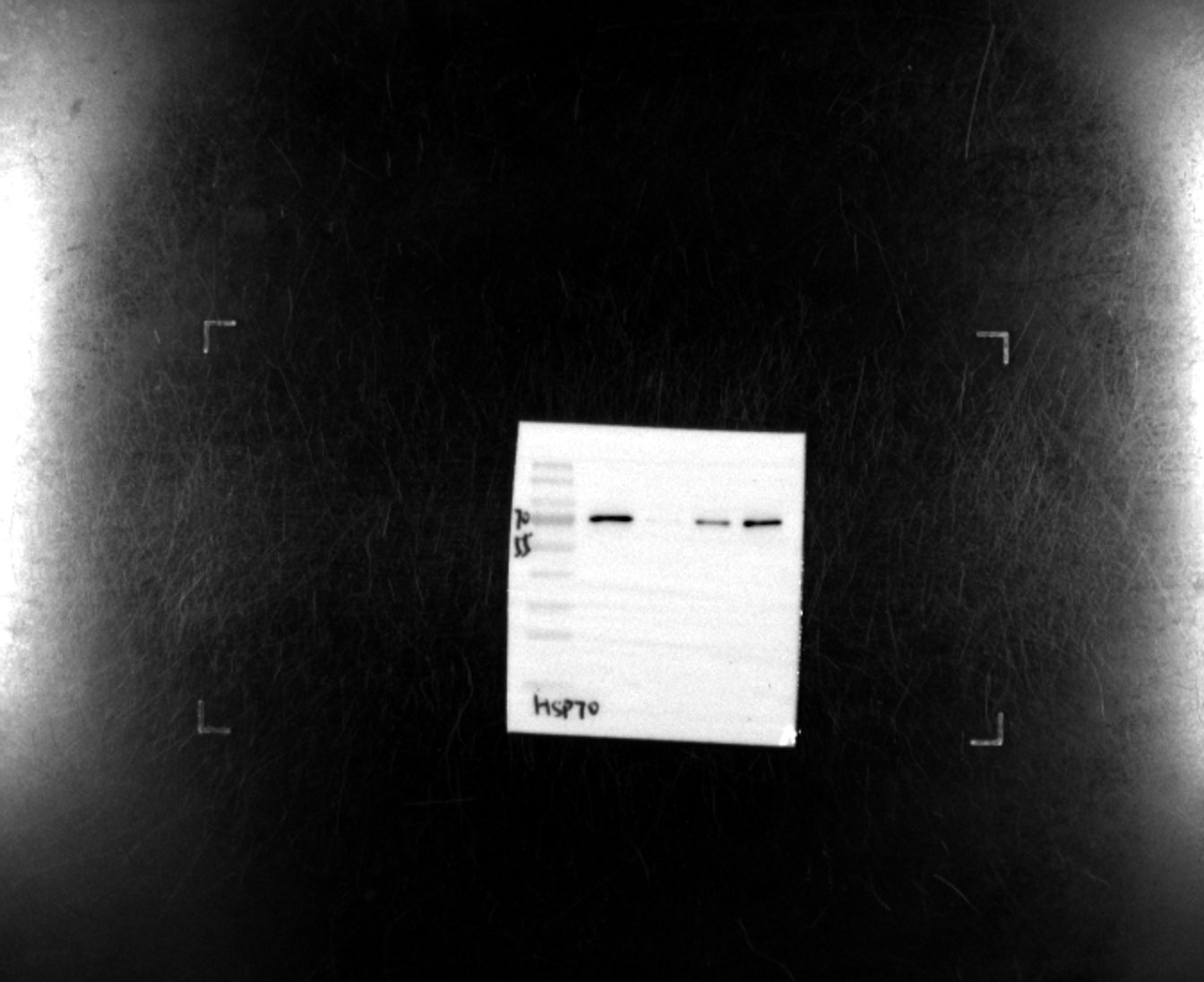


5E-HSP70


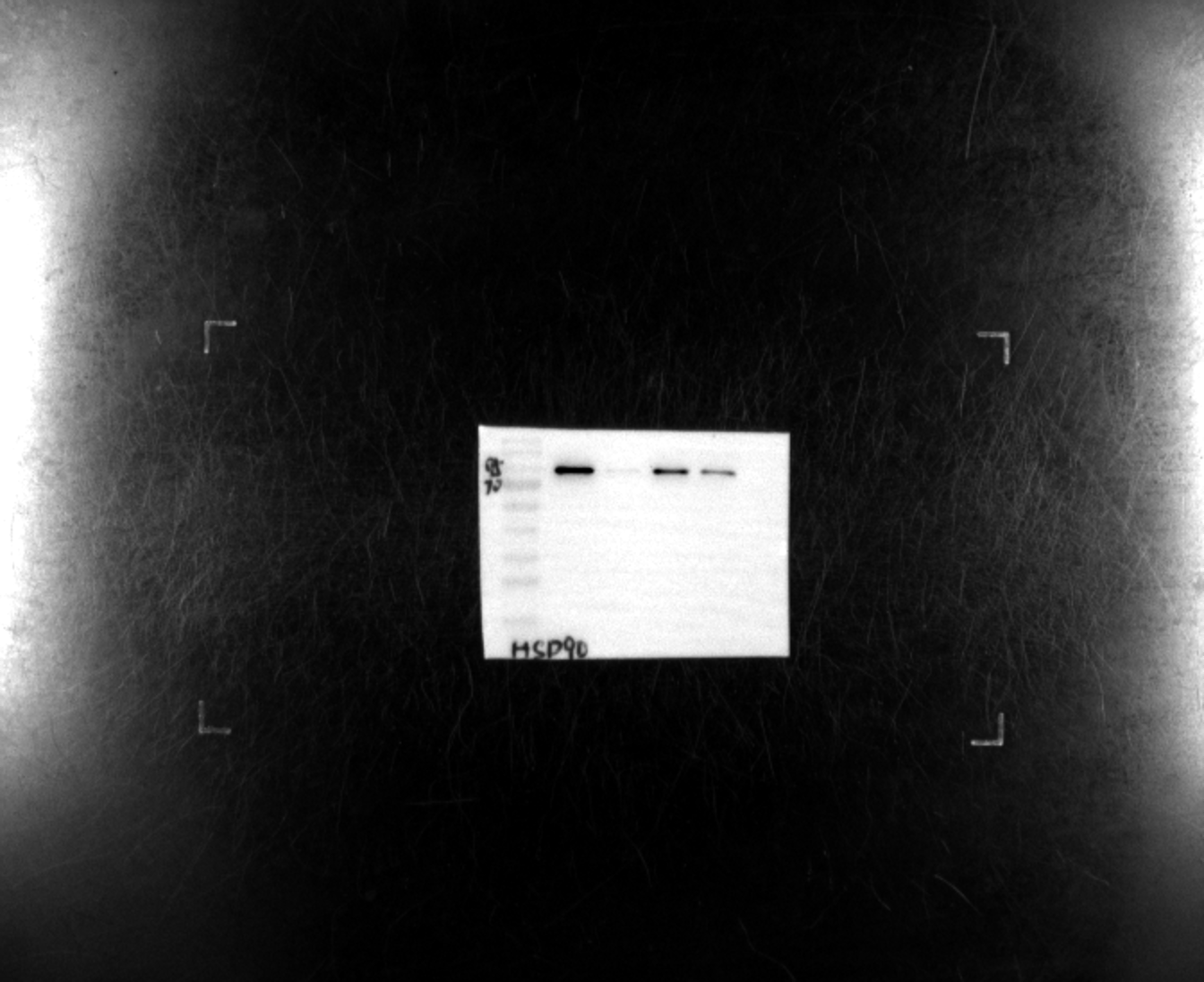


5E-HSP90


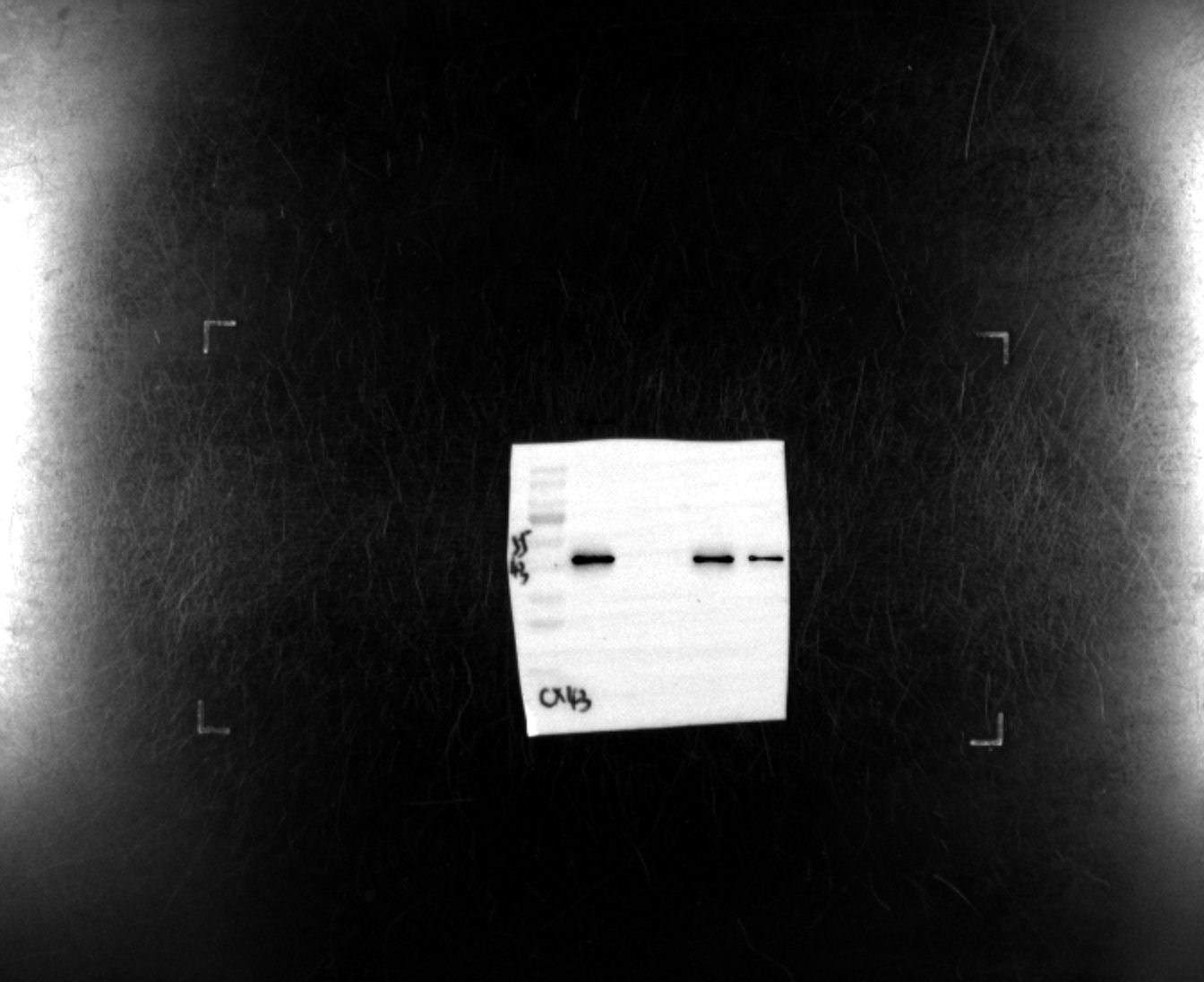


5F-CX43


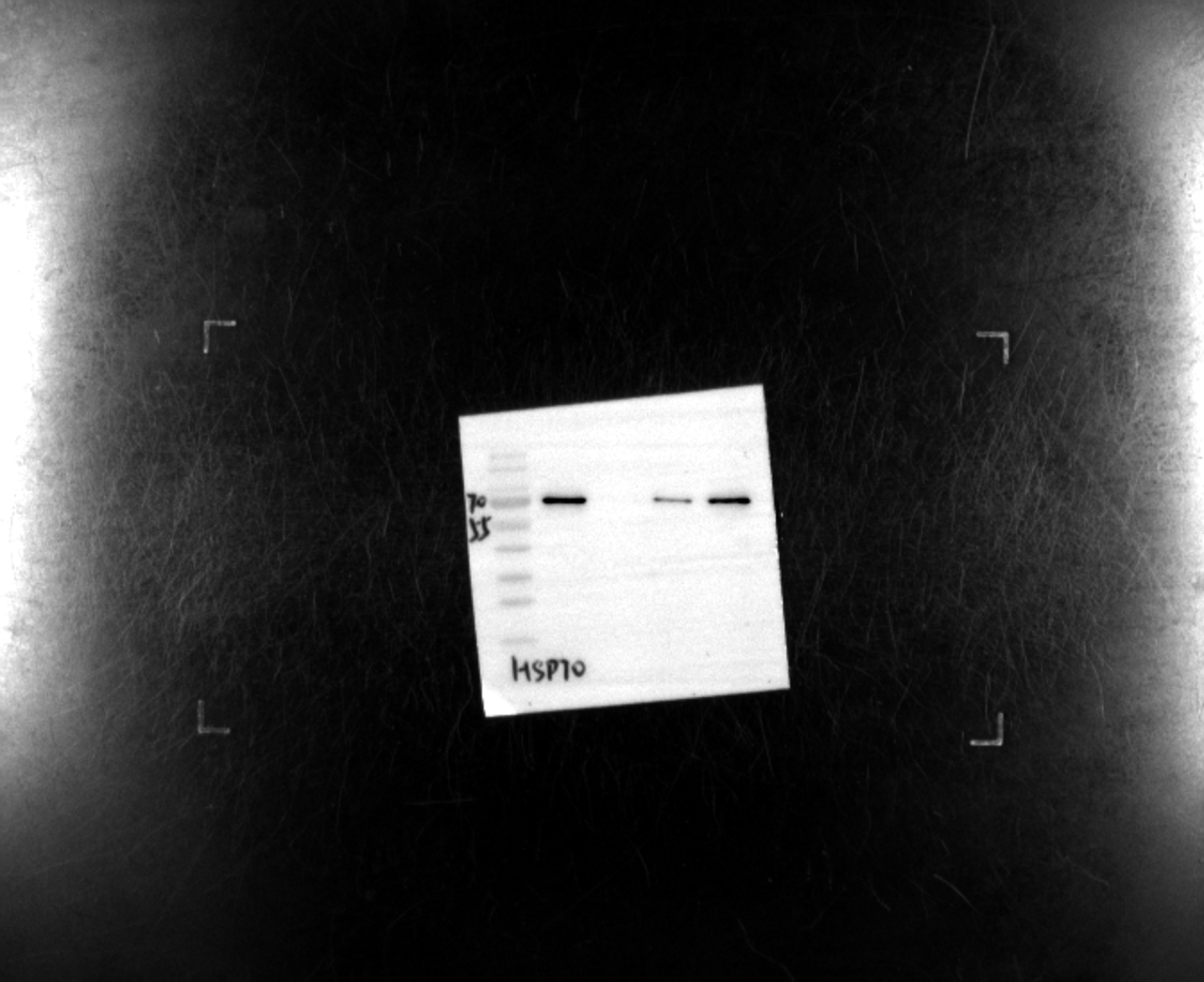


5F-HSP70


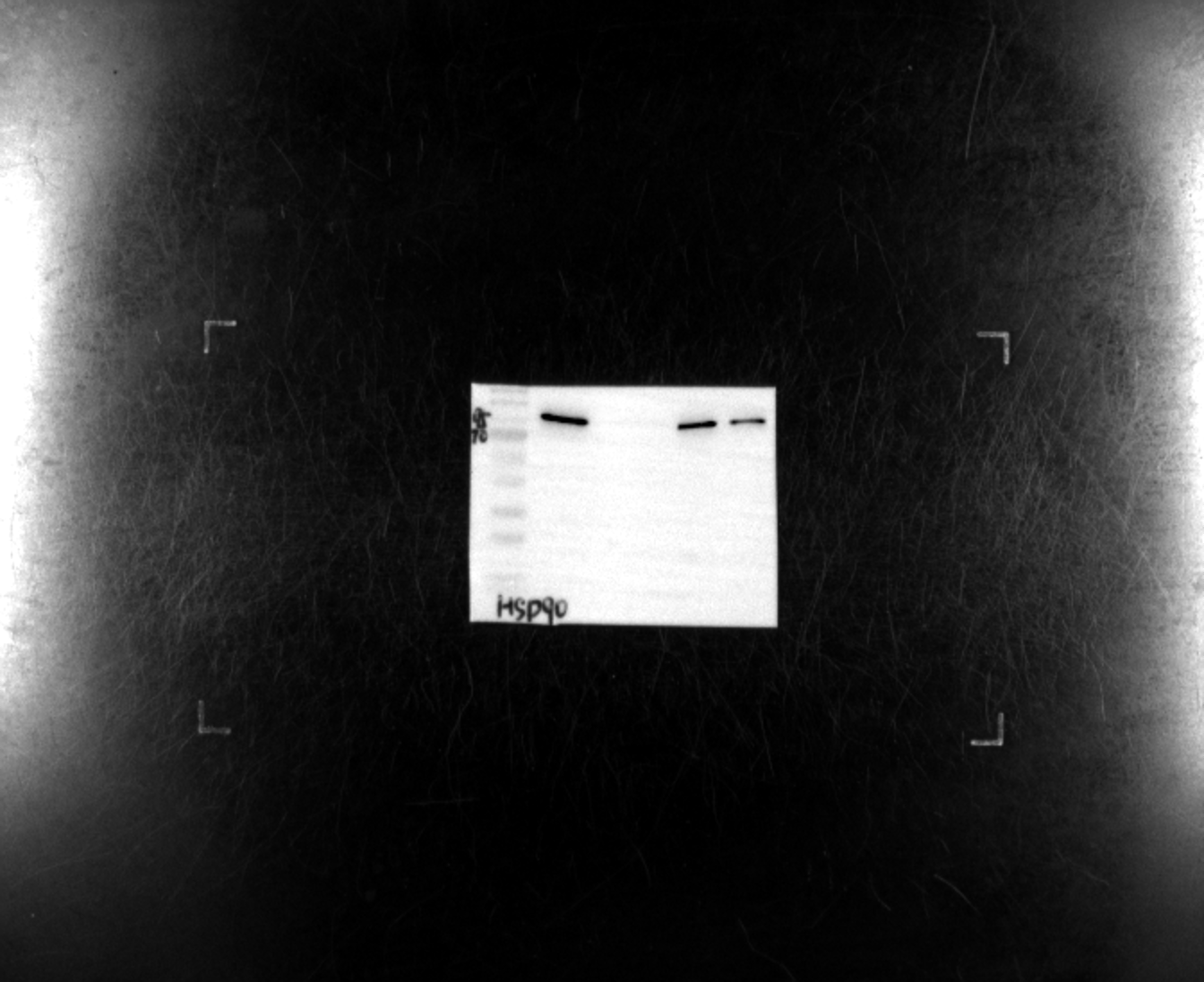


5F-HSP90


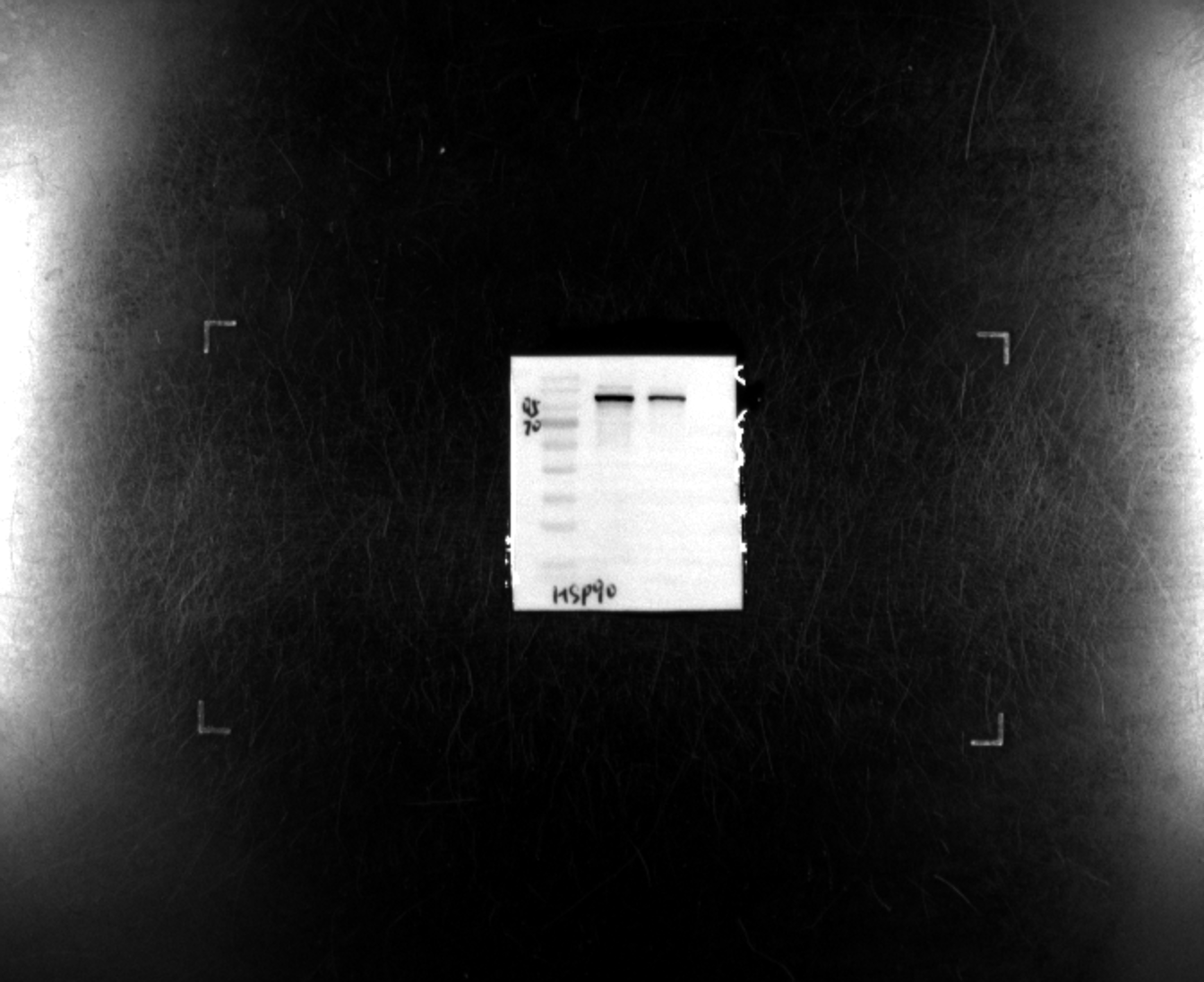


6A-1-HSP90


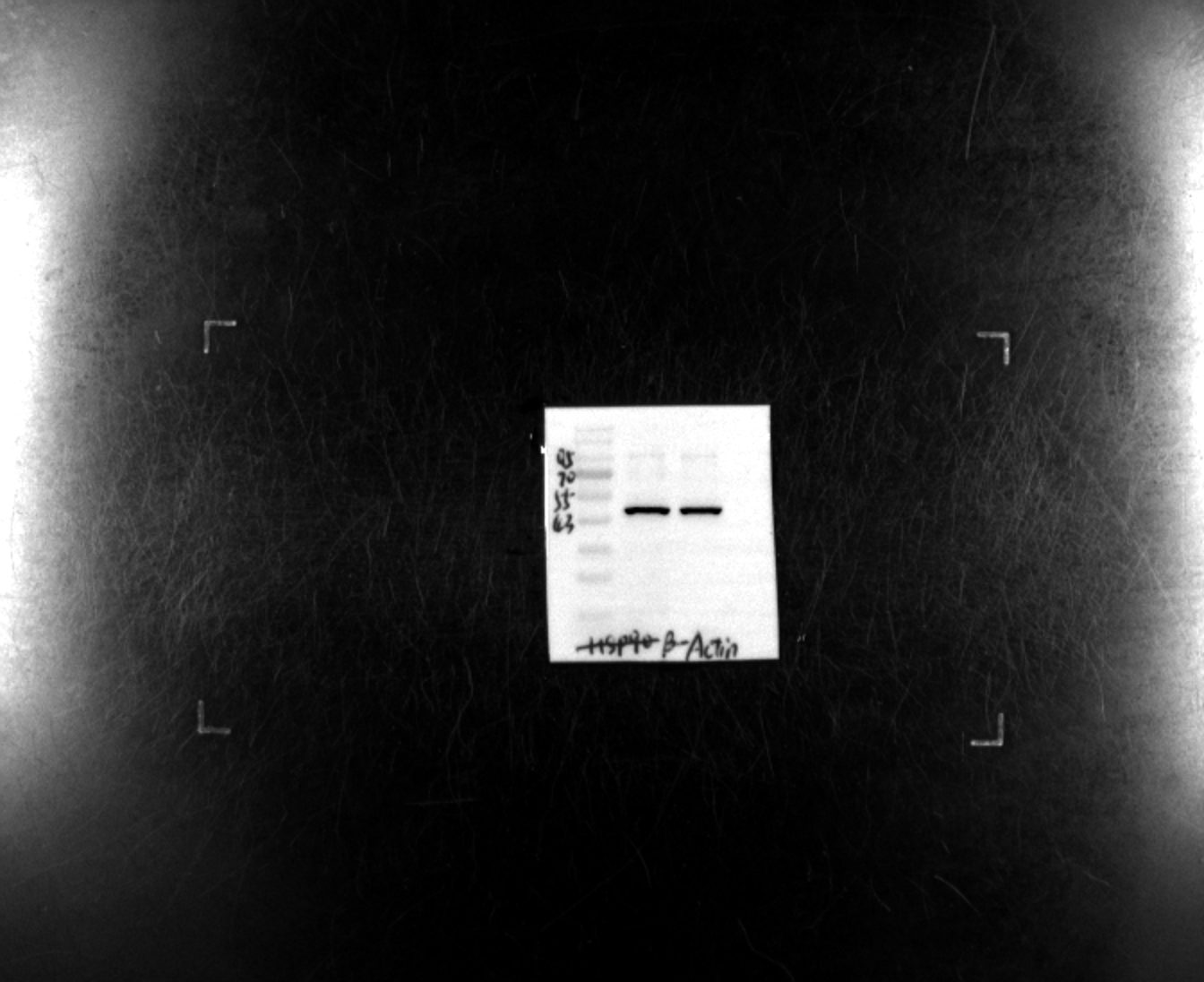


6A-1-β-ACTIN


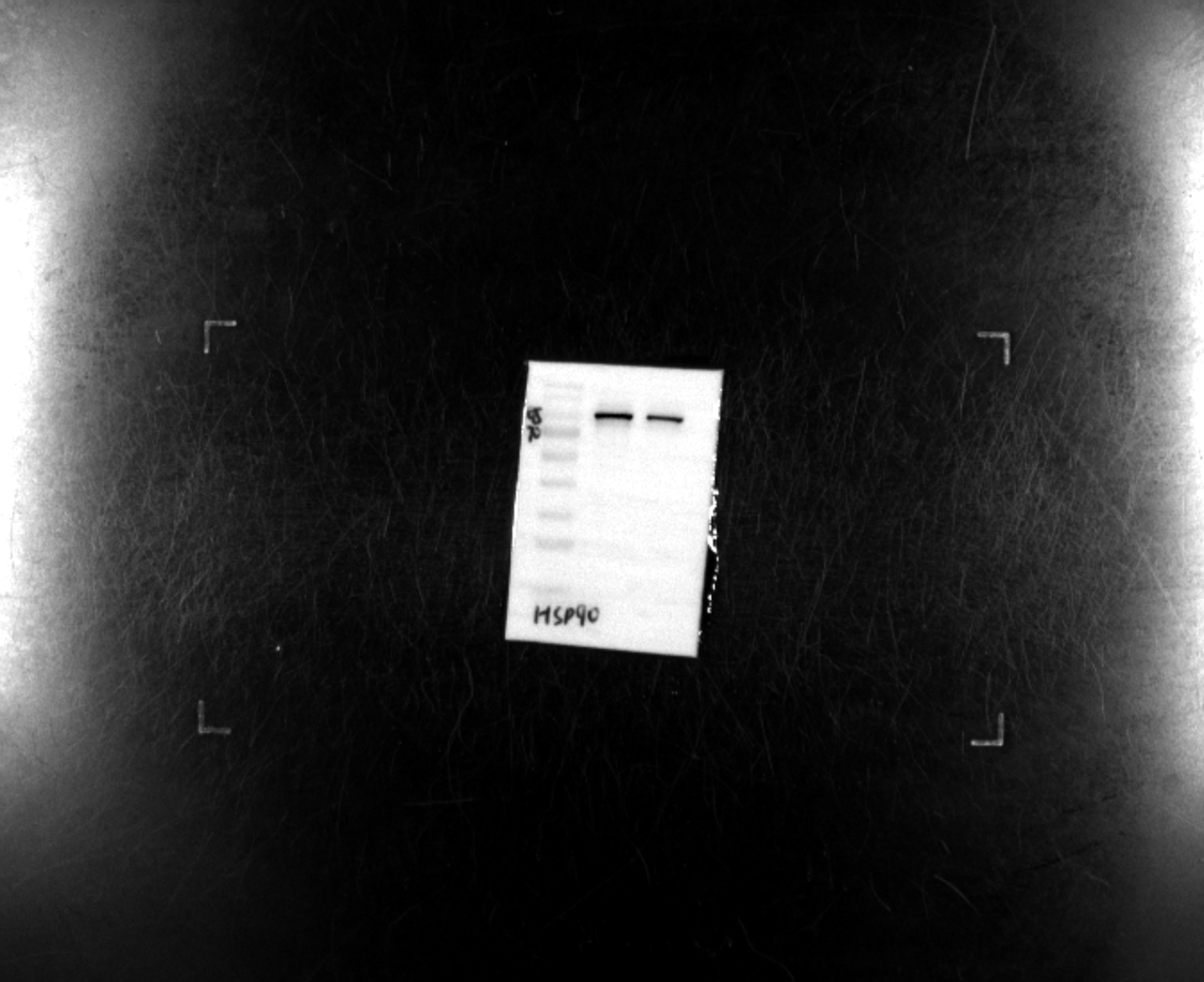


6A-2-HSP90


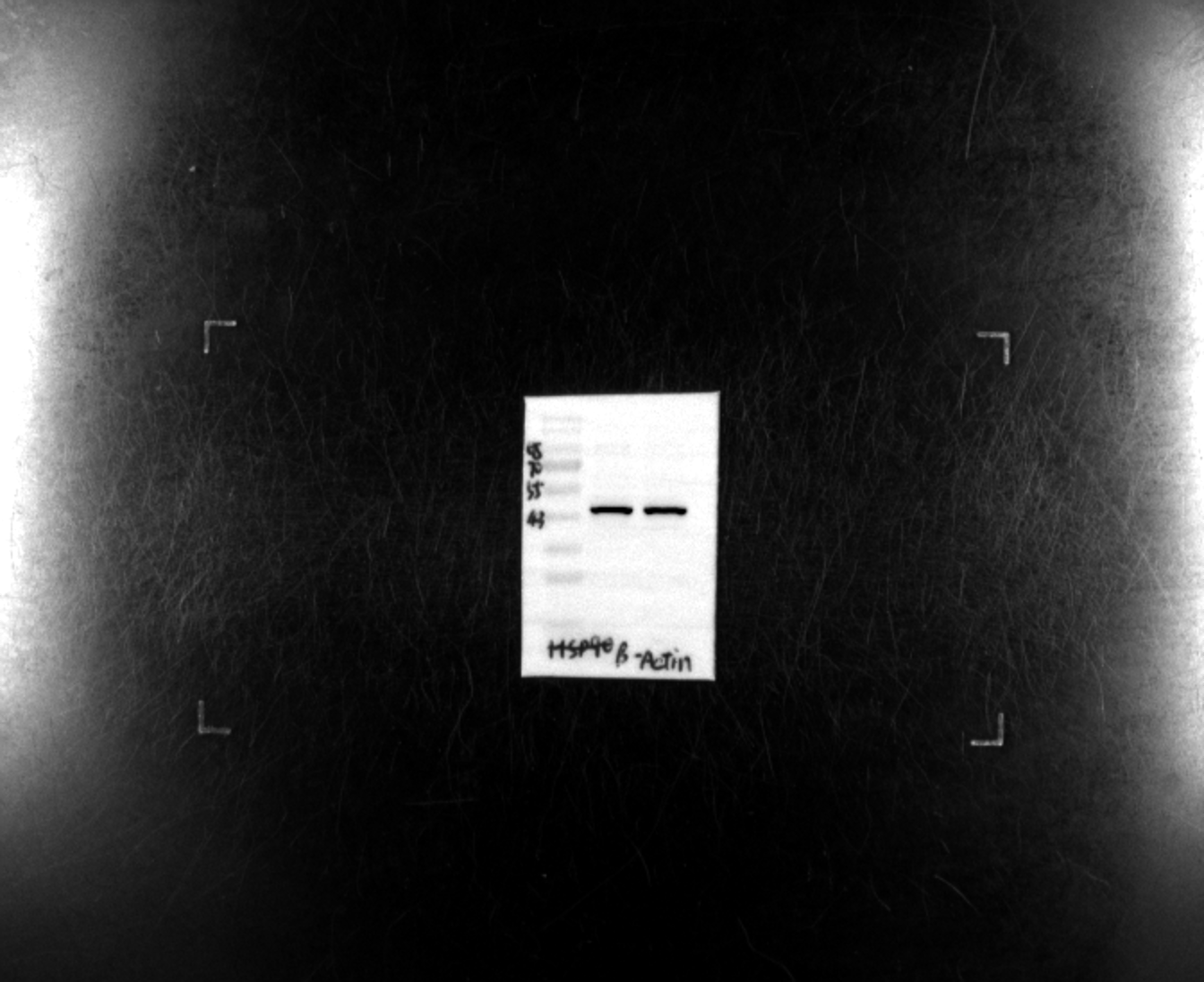


6A-β-ACTIN


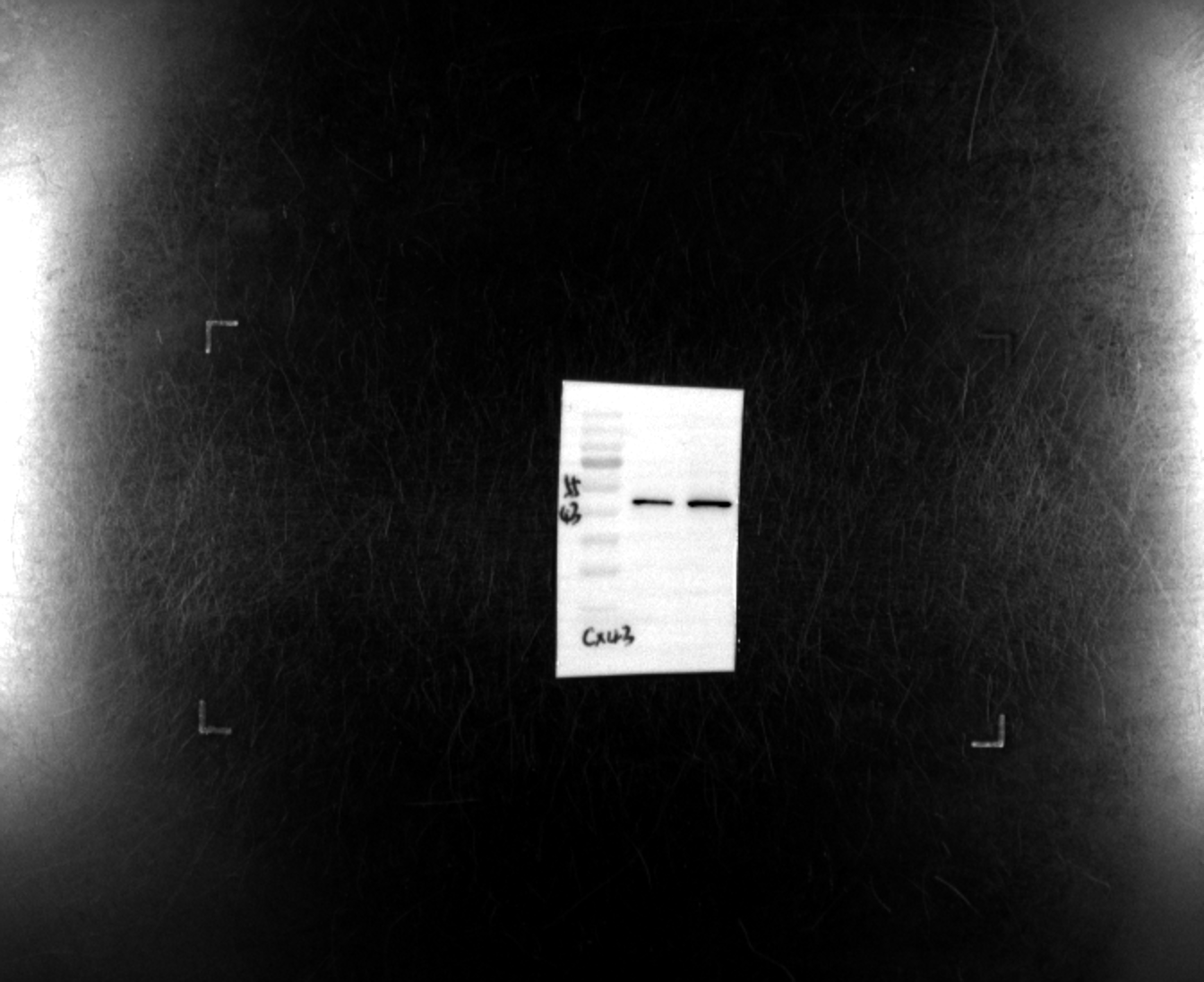


7B-CX43


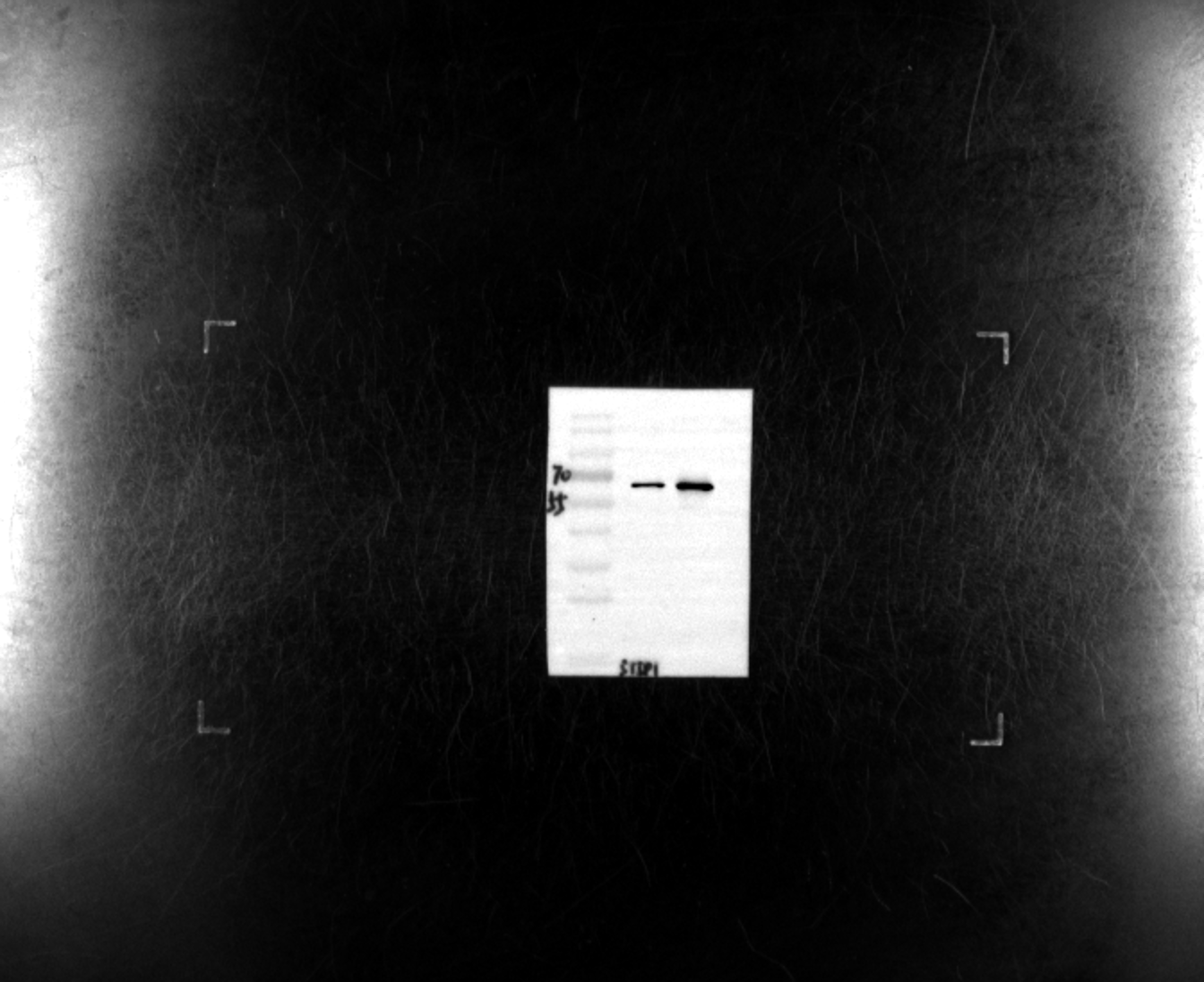


7B-STIP1


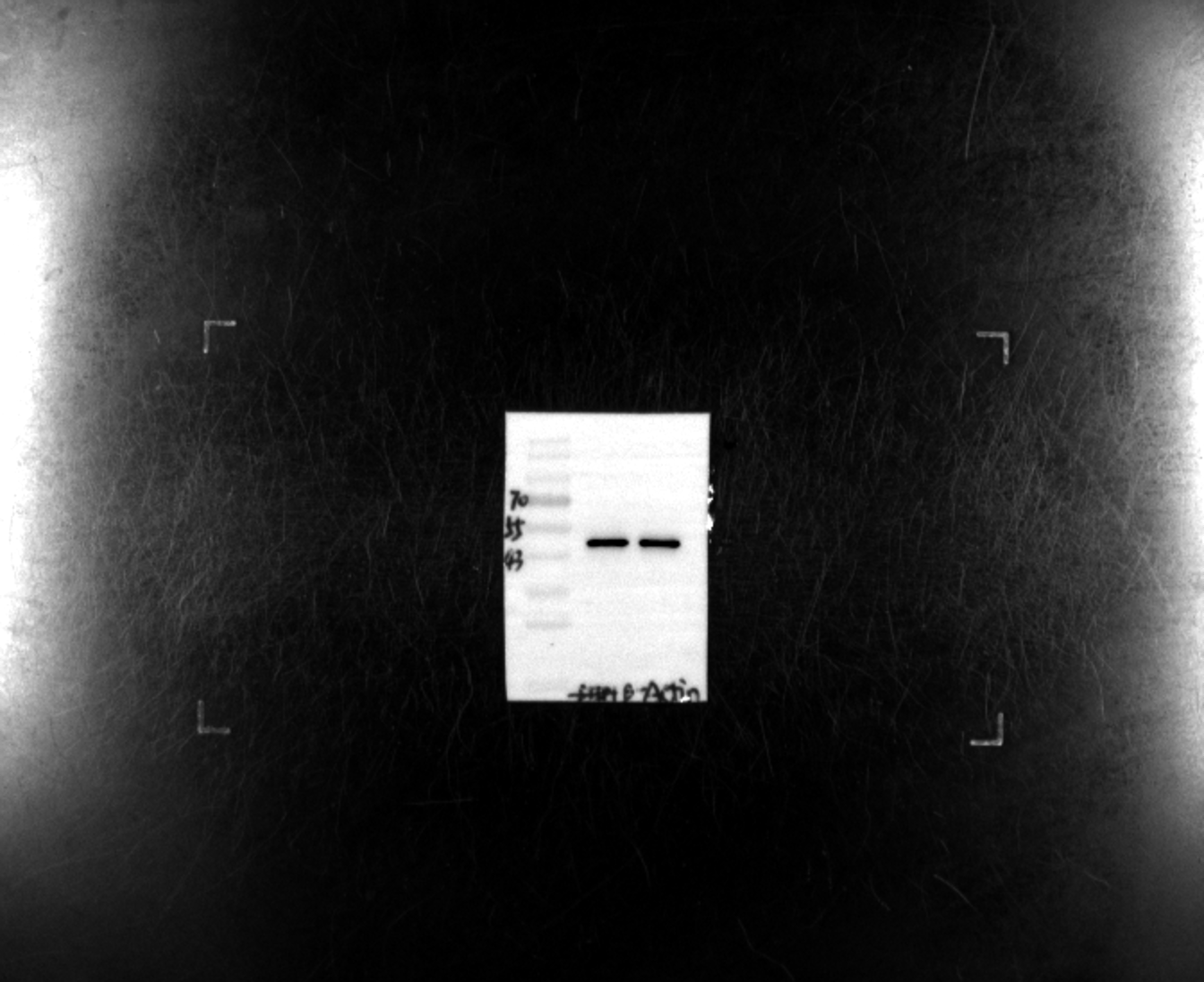


7B-β-ACTIN
